# Cardiac function in zebrafish embryos is linked to an androgen receptor-adrenomedullin-proepicardium axis

**DOI:** 10.1186/s12964-026-03107-4

**Published:** 2026-07-30

**Authors:** Max Duong Phu, Alessandra Guerrero Samanidis, Sabrina Laibacher, Martina Burczyk, Yara Alkhars, Ana Janovic, Ashraf Al Madhoun, Ilona S. Skerjanc, Martin D. Burkhalter, Melanie Philipp

**Affiliations:** 1 Department of Experimental and Clinical Pharmacology and Pharmacogenomics, Section of Pharmacogenomics, Faculty of Medicine, Eberhard-Karls-University Tübingen, Wilhelmstrasse 56, Tübingen, 72074 Germany; 2 Institute of Biochemistry and Molecular Biology, Ulm University, Albert-Einstein- Allee 11, 89081 Ulm, Germany; 3 Genetics and Bioinformatics, Dasman Diabetes Institute, Jasim Mohamad Al Bahar St, Dasman 15462 Kuwait City, Kuwait; 4 Department of Biochemistry, Microbiology, and Immunology, Faculty of Medicine, Ottawa University, 451 Smyth Rd, Ottawa, ON K1H 8M5 Canada

**Keywords:** Androgen receptor, Congenital heart defects, Heart development, Adrenomedullin, Proepicardium

## Abstract

**Background:**

Congenital heart defects (CHDs) comprise the most common congenital malformation affecting nearly 1% of all newborns. In individuals with sex chromosome aneuploidy syndromes, however, the prevalence reaches up to 50% of all livebirths. One commonality to these syndromes is a marked reduction in sex hormones, particularly androgens. Androgen receptor (Ar) insufficiency represents a culprit in the pathophysiology of adult onset cardiovascular disease and arrhythmia, but there are no reports regarding Ar function during heart development. This is surprising as androgens along with its nuclear receptor exist already during early development, even before gonads develop and become functional.

**Methods:**

We evaluated the role of the Ar during vertebrate heart development by generating loss-of function in zebrafish embryos via pharmacological inhibition, transient CRISPR/Cas9 treatment, or interference of splicing as well as murine cell culture.

**Results:**

Attenuation of Ar function in zebrafish embryos prevents normal cardiac morphology and physiology as apparent by edema, bradycardia and arrhythmia. Molecularly, we identified increased abundance of adrenomedullin (Adm) 2a as a likely cause for the observed defects. Cellularly, these phenomena may be linked to impaired formation of proepicardial cells, which are reported to intermingle with cells of the conduction system and influence cardiac pacing.

**Conclusions:**

A disrupted Ar – Adm2 axis possibly contributes to the increased frequency of CHD in individuals suffering from sex chromosome aneuploidy syndrome.

**Supplementary Information:**

The online version contains supplementary material available at 10.1186/s12964-026-03107-4.

## Abstract (plain language summary)

Testosterone function is generally mediated by androgen receptors in the nucleus of the cell. Interestingly, the androgen receptor exists already in early embryos, long before sexual maturation will be initiated. This study aimed to investigate the function of the androgen receptor during heart development using zebrafish embryos. Embryos lacking functional androgen receptors display severely impaired heart development resulting in a slower and irregularly beating heart. Consistently, several important structures of the heart including the conduction system necessary for cardiac rhythmicity fail to form and function properly in the absence of androgen receptor function. Loss of androgen receptor function leads to elevated expression of adrenomedullin 2a, which has previously been implicated in heart development and function. Consistent with a study reporting adrenomedullin 2a originating from the proepicardium loss of proepicardial genes could be observed in the absence of the androgen receptor. Taken together, this study identifies a novel role of the androgen receptor during early heart development, most likely independent of the later biological sex.

**Supplementary Information:**

The online version contains supplementary material available at 10.1186/s12964-026-03107-4.

## Background

Congenital heart defects (CHD) are the most common inborn malformations [1] and comprise a group of morphological cardiac anomalies which develop during embryonic development. Many different types and degrees of structural defects (i.e. septal defects, transposition of great arteries and many others) can occur and many of these eventually impact cardiac physiology. Genetically, a growing number of variations could be associated with CHD. However, we still lack a complete picture of all genes guiding proper heart development, not least, because the same gene variant may precipitate in different types of CHDs, possibly due to environmental influences and the life style as well as health status of the expecting mother [2]. Moreover, a bouquet of several genetic variations may be necessary to interfere with normal heart development [3, 4].

Some of the highest prevalences for CHDs are carried by individuals with chromosomal abnormalities. In patients with sex chromosome aneuploidy up to 50% of all newborns suffer from CHD [5]. A common condition presenting particularly in sex chromosome anomalies such as Klinefelter syndrome and Turner syndrome is a reduction in testosterone levels [6, 7] suggesting reduced androgen receptor signaling as a potential cause for CHDs.

Androgens mediate their effects mainly through two receptors. The canonical androgen receptor (Ar) is a nuclear receptor serving as a transcription factor upon binding its ligand. In addition, there is a membrane receptor likely resembling a G protein-coupled receptor named Gprc6a (G protein-coupled receptor, class C, group 6, member A), which has recently been associated with heart function during development [8]. In the adult, an increased risk for cardiovascular disease and arrhythmia has been observed in the absence of nuclear Ar-induced signaling. For instance, Ar antagonist treatment during prostate cancer therapy may result in QT elongation [9, 10]. In the early embryo, however, even before gonads have developed, the impact of Ar signaling on the heart remains uncharacterized. This is surprising as the Ar has been detected in cardiac tissues already very early during development [11–13] and Ar knockout mice have been reported to develop smaller hearts than their wild-type littermates [14]. Moreover, zebrafish embryos treated with an Ar inhibitor displayed impaired heart function including arrhythmia [15]. Here, we performed a detailed analysis of Ar function during zebrafish heart development.

Zebrafish embryos comprise a simple, yet elegant model for the study of vertebrate heart development. The zebrafish heart becomes functional from 24 h post fertilization (hpf) on and soon after compartmentalizes into two chambers, a single atrium and one ventricle [16]. The zebrafish cardiac conduction system (CCS) with the sinoatrial node (SAN) and the atrioventricular canal (AVC) is well conserved compared to other vertebrates and as in other species, abnormal development or gene expression in either of these two structures can result in irregular pacing of the heart [17]. Importantly, SAN and AVC defects and with that arrhythmia may not only be due to cellular or molecular changes within the CCS components, but can also be caused by cells in close contact with SAN and AVC. These include other cardiac cells, such as those originating from the proepicardial organ, which appear to intermingle with CCS cells and are essential for normal cardiac electrogenic function [18].

In this study, we present that interfering with normal nuclear Ar function severely impairs cardiac functionality during vertebrate development before the gonads have become functional. We observed structural defects of the developing heart, which were accompanied by impaired contractility and rhythmicity. Molecularly and cellularly, we identified elevated *adrenomedullin 2a* (*adm2a*) levels and a loss of proepicardial markers as culprit. Abrogation of Adm2a or induction of proepicardium development through mild overexpression of bone morphogenetic protein 2b (Bmp2b) restored proepicardial marker expression and cardiac functionality. We hence conclude that androgen signaling regulates early heart development and function at least partially by dampening Adm2a expression and facilitating proepicardium formation.

## Methods

### Sex as a biological variable

In this study, zebrafish embryos were used before a defined biological sex had developed.

### Inhibitors

Adrenomedullin 22–52 was purchased from MedChemExpress, ARN-509 [19] and flutamide [20] were from Selleckchem, and PF-998,425 [20], tolterodine [21] and tricaine [22] were obtained from Sigma-Aldrich. All guide RNAs along with HiFi-Cas9 were purchased from IDT.

### Cloning

For the generation of in situ probes, plasmids were cloned using the TOPO TA cloning kit (Thermo Fisher) for the following genes: *adm2a* (Genbank: NM_001130135.1, 439 bp fragment), *receptor activity modifying protein 2* (*ramp2)* (Genbank: NM_001114432.1, 500 bp fragment), *transcription factor 21* (*tcf21*) (Genbank: NM_001037681.2, 969 bp fragment), *wt1a* (Genbank: NM_131046, 1038 bp fragment). Similarly, a portion of the *ar* to detect its expression amplified as described in Gorelick et al. [23] and cloned by TOPO TA cloning. All other probes have been used and described previously [24, 25].

In order to prepare capped RNA, the open reading frames of zebrafish *ar* (Genbank NM_001083123.1) and *bmp2b* (Genbank: NM_131360.2) were amplified from zebrafish cDNA using Q5 polymerase (NEB) and cloned into pCS2+ via ClaI / StuI and BamHI / EcoRI, respectively. To allow for immunofluorescence and immunoprecipitation, a Flag-tag was added to the N-terminus of zebrafish Ar.

Luciferase constructs for reporter assays were cloned by amplifying a 1970 bp and a 1471 bp long sequence upstream of the transcription start site of the *adm2a* gene and insertion into the promoterless luciferase plasmid pGL4.10 (luc2) (Promega) via NheI and KpnI.

### Generation of capped RNA

The SP6 mMessage mMachine Kit (Thermo Fisher) was used to in vitro transcribe capped RNA from NotI-linearized plasmids. The RNA was then phenol-chloroform-purified and precipitated using ethanol and ammonium acetate. The RNA pellet was dissolved in ultrapure water and stored at -80 °C until further use.

### Zebrafish maintenance and manipulation

Adult zebrafish were maintained under standardized conditions essentially as recommended in [26] in a Zebtec tank system (Tecniplast) in our fish facility. Health status was monitored daily. Wild-type fish (AB or Ekwill) as well as transgenic fish expressing GFP under a cardiomyocyte-specific promoter (Tg(myl7:GFP)) [27] were used. Fertilized eggs for manipulations were obtained by natural mating. No adult zebrafish was euthanatized for the experiments in this study. For all experiments, eggs of a clutch were equally and randomly distributed into different treatment groups. Blinding was not performed as phenotypes were too obvious to allow for efficient blinding. Confounders were not controlled. Experiments were repeated at least three times with embryos from different clutches. Please, see figure legends for exact numbers of embryos used in each experiment, controls, and compared groups. Unfertilized eggs as well as dead and profoundly developmentally retarded embryos were excluded, yet not counted.

Ar loss-of-function (LOF) was either obtained by treatment with pharmacological inhibitors from tailbud stage on or through microinjection of a splice blocking antisense morpholino oligonucleotide (MO) (Gene Tools) targeting the intronic sequence right upstream of the third exon of the *ar* (5’- CTATAGGAGAAGAAACAGACAGTCT). Microinjections were done at the 1–2 cell stage as described previously [28] and controlled against injections with a standard control MO (Gene Tools) and uninjected wild-type embryos. To rescue Ar morphants, fertilized eggs were consecutively injected with splice blocking MO and capped RNA. Ar promoter crispants were generated with the following guideRNAs (gRNA): 5’- UAUGGGAAAUAUGACUGACU, 5’- UGGUCGUUAUGGUGCACCCA and 5’- AUGCUCAGAUUUAACCUUAC. Ar exon crispants were generated by injection of three gRNAs assembled into one ribonucleoprotein targeting the following sequence in exon 1: 5’-CAAGAAGTGCGGCTGCCTAC, 5’-TGTCCGTATCTTTGGGCTTA and 5’-CATGACCCATTATGCCCACC. Adm2a LOF was achieved by transient Crispr/Cas9-mediated gene editing and gRNAs targeting the following sequences: 5’-CAACTGCTGGCTCTACCGGT and 5’-GTCCACCGTAACACTCGAGA. Alternatively, a peptide inhibitor was administered with the embryo medium from tailbud stage on (1 µM final concentration). All gRNAs (IDT) were assembled into ribonucleoprotein complexes with recombinant HiFi Cas9 (IDT) according to a protocol published on the IDT webpage. Phenol Red (Sigma) was added to monitor the injection.

### Splice blocking verification by reverse-transcription-PCR (RT-PCR)

20 embryos (24 hours post fertilization (hpf)) were dechorionated and total RNA was isolated using the Quick-RNA Miniprep kit, which includes a DNaseI step for genomic DNA removal (Zymo Research). Equal amounts of RNA were reversely transcribed into cDNA using the Protoscript II Kit (New England Biolabs) and used in a PCR amplifying a 436 bp product from exon 2 to exon 4 of zebrafish androgen receptor (Forward: 5-CCTGCCTAATCTGCTCTGATG; Reverse: 5’-TCGTTAAGGCTGGTGAGAAGA). MO-mediated splice blocking resulted in an additional smaller band due to skipping of exon 3. As control, zebrafish actin 1 was amplified (Forward: 5-GACATCAAGGAGAAGCTGTGC; Reverse: 5’-CACTTCCTGTGAACGATGGAT).

### Crispr/Cas9 verification

A 1080 bp sequence upstream of the transcription start site of the *ar* was amplified from genomic DNA of injected embryos to verify genome-editing of the promoter of the *ar* (Fw: 5’- AGCAAAGCCAAGAGAGATCAAAT, Rev: 5’- GCACGCGTGTTATCCATAAATGA) (Figure S6). Alternatively, a fragment of exon 1 was amplified to assess editing of guide RNAs targeting exon 1 of *ar* (Fw: 5’- ATGGAGGTTCCGGTCGGG, Rev: 5’- GTGGCCAGTAGCATAAGGTGT) and a fragment stretching form exon 2 to exon 3 of *adm2a* was amplified to assess editing by *adm2a* guideRNAs (Fw: 5’- CTCTTCAAACAGCACAATACCCTG, Rev: 5’- TGGTCTTGGTTTTAGCCTCTAGAC). Fragments were gel-extracted and Sanger sequenced. In the case of Ar exon crispants editing efficiency was assessed using TIDE [29]. gRNA1 and 2 targeted against *ar* resulted in editing efficiencies up to 21% and 79%, respectively. Substantial gene editing efficiency could not be detected by gRNA3 in our hands. Gene editing of *adm2a* resulted in deletion of the sequence between the two gRNA binding sites, which produced a premature stop codon.

### Analysis of cardiac performance

Heart rates were manually counted at 26 °C and with the help of a brightfield stereomicroscope. Irregular beating of either chamber as well as atrioventricular blockades as detected by measuring atrial and ventricular beating rates separately were collectively considered arrhythmia. In addition, silent atrium and silent heart were counted as arrhythmic, too.

A previously described heart analysis software (Viewpoint, France) was used to determine the ejection fraction and corrected Qt (Qtc) interval based on fluorescence high-speed movies of beating hearts obtained from Tg(myl7:GFP) embryos [30]. The Cardiac Performance Software calculated movies of fluorescently labeled zebrafish hearts into M-mode like pictures, which allowed for measurement and calculation of several cardiac parameters such as the chamber-specific ejection fraction or the corrected Qt time.

### Cell culture of HEK293T cells

HEK293T (ATCC) cells were cultivated in DMEM containing 10% fetal bovine serum and 1% penicillin/streptomycin (all Thermo Fisher) in a humidified incubator at 37 °C and 5% CO_2_. Charcoal-stripped serum was used to culture cells in the absence of steroid hormones, which may normally be contained in fetal bovine serum. Transfections were achieved using Lipofectamine 3000 and Opti-MEM (both Thermo Fisher). To analyze subcellular localization of the zebrafish Ar, 24 h post transfection cells were transferred to cover slips coated with poly-D-lysine (Sigma-Aldrich) and fixed after stimulation with dihydrotestosterone (DHT) for 10 min with 4% buffered paraformaldehyde (Sigma-Aldrich) at room temperature.

### Whole mount in situ hybridization (WMISH)

Whole mount in situ hybridization was done essentially as previously described [31] using DIG-labeled antisense riboprobes. Plasmids for generation of *notch1b* and *T-box transcription factor 8* (*tbx8*) probes as well as *hcn4* and *T-box transcription factor 2b* (*tbx2b*) were kindly provided by Ken Poss and Jeroen Bakkers, respectively. The *tcf21* probe was synthesized as reported in Serluca et al. [32].

### Immunofluorescence

After fixation, HEK293T cells seeded on cover slips were permeabilized for 10 min using 0.1% tritone X-100 in phosphate-buffered saline (PBS), blocked with 3% bovine serum albumin (BSA, Sigma-Aldrich) in PBS containing 0.1% Tween-20 (PBST) and incubated for 2 h at room temperature with mouse anti-Flag M2 antibody (Sigma-Aldrich, catalog no. F3165, 1:1000). After three washes with PBST, the coverslips with cells were incubated for one hour with Alexa 488-coupled secondary antibody (Thermo Fisher, 1:1000), washed again and mounted using Vectashield containing Dapi (Vector Labs). Edges were sealed using nail polish.

For sarcomere visualization, zebrafish were fixed in 1% formaldehyde (Sigma-Aldrich) for 50 min at room temperature. After washing with PBS, embryos were blocked for 1 h at room temperature using blocking buffer containing 10% normal goat serum (Biozol), 2 mg/ml BSA, 0.2% saponin (Sigma-Aldrich) in PBS. Next, embryos were incubated with mouse anti-S46 (DSHB, 1:100) diluted in blocking buffer at 4 °C overnight. After three washes with PBS containing 0.2% saponin, embryos were incubated with Alexa 488-coupled secondary antibody (Thermo Fisher, 1:1000) for several hours at room temperature, washed again and mounted between cover slips sealed with vacuum grease using Vectashield containing Dapi (Vector Labs).

### Imaging

Imaging of live embryos (for immobilization anesthetized by immersion in 0.02% (m/v) buffered tricaine) and such processed by WMSIH was accomplished with a Leica 125 stereo microscope and either an IC80HD or a Flexacam C1 color camera. Videos of beating hearts for automated cardiac performance analysis were obtained with a Leica M205 FCA microscope equipped with a Leica DFC9000GT sCMOS camera by selecting a small region of interest and at a rate of 100 frames per second for 10 s. Zebrafish hearts and HEK293T cells processed by antibody staining were imaged using a Leica Stellaris 5 confocal microscope using glycerol immersion.

### P19 cells differentiation into cardiomyocytes

P19 cells (CRL-1825, ATCC) with reduced AR expression, termed P19[shAR] or Scrambled controls, termed P19[shScrambled] were previously generated as described in [11]. The cells were essentially cultured and differentiated as described [11] with the following modifications. First, the cells were dissociated into single cells and resuspended in the differentiation media (α-MEM) supplemented with 10% fetal bovine serum, 0.8% Dimethyl sulfoxide). Then, for the generation of embryonic body structures, 1.8 × 10^6^ cells were transferred to a well of the eight-well AggreWell Plate (Stem Cell Technologies). To enhance cardiac differentiation, cells were treated with either 10 µM 5-Azacytidine (cat. no. A2385, Sigma-Aldrich) or 10 µM Oxytocin (cat. no. O4375, Sigma-Aldrich). After 24 h incubation period at 37 °C in a 5% CO_2_ incubator, the embryonic bodies were transferred to Petri dishes and maintained in differentiation media supplemented with 5-Azacytidine or Oxytocin. On day 5, embryonic bodies were plated as monolayers without drugs and harvested as indicated.

### Quantitative Polymerase Chain Reaction (qPCR) analysis

Total RNA was extracted with Qiagen’s RNeasy kit (for cells) or Zymo Research’s RNA Microprep Kit including treatment with DNAse I (for pools of manually dechorionated zebrafish embryos). Equal amounts of RNA were transcribed into cDNA with the High-Capacity cDNA Reverse Transcription Kit (Applied Biosystems, used for cells) or the Protoscript II Kit (NEB, used for zebrafish RNA). Zebrafish hearts were isolated as described before [15] and cDNA prepared directly with the Taqman Gene Expression Cells-to-Ct kit (Thermo Fisher) with the inclusion of a DNaseI treatment. Here, 20–60 isolated hearts were used per isolation and reverse transcription. qPCR was done in triplicates either using SYBR green on a 7500 Fast Real-Time PCR System (Applied Biosystem) or with NEB’s Luna Universal Probe Master Mix and the Universal Probe system (Roche) on a Lightcycler 480 II instrument (Roche) or a QuantStudio 3 thermal cycler (Applied Biosystems). Generally, intron-spanning assays were designed.

Further information regarding qPCR assays (primers and probes) can be found in the supplement (Table S1).

### Western blotting

HEK 293T cells that were transfected with pCS2+-FLAG-zfAR or empty vector and 24 h later mechanically dislodged, were washed with PBS and lyzed in SDS lysis buffer (2% SDS, 100 mM Tris pH 6.8), which contained proteinase and phosphatase inhibitors (cOmplete protease inhibitor EDTA free and PhosSTOP™ Phosphatase Inhibitor, Roche). A BCA assay (Sigma-Aldrich) was applied to assess protein contents. Equal amounts of proteins were separated on a 4%-12% Bis-Tris Bolt gel with MES Bolt running buffer (both Invitrogen) and Precision Plus Protein Kaleidoscope Prestained Protein ladder (Bio-Rad), followed by transfer onto a nitrocellulose membrane (pore size 0.2 μm, Bio-Rad). Staining with ponceau S (Sigma-Aldrich) allowed for guided cutting of membranes, which in the following were blocked with 3% milk in tris buffered saline containing 0.5% Tween-20. Antibodies used were mouse anti-FLAG M2 (catalog no. F3165, Sigma), mouse anti-GAPDH (catalog no. MAB6374, Millipore) and donkey anti-mouse IgG-Alexa 800 plus (Thermo Fisher) (all 1:1000) and detected in an iBright 1500 FL imaging system (Thermo Fisher).

### Chromatin immunoprecitation (ChIP)

Sub-confluent HEK293T cells were transfected with pCS2+-FLAG-zfAR using the Lipofectamine 3000 system (Invitrogen). The next morning, cells were expanded into three 10 cm culture dishes. In the evening, culture medium was exchanged to starvation conditions (DMEM with 1% penicillin / streptomycin). After three hours stimulation with 1 nM dihydrotestosterone (DHT, Sigma-Aldrich), media was removed and cells crosslinked during 13 minutes with 1 ml 1% formaldehyde in PBS at 37 ˚C, followed by quenching with glycine (125 mM final concentration) during 5 minutes at room temperature. Cells were washed with ice-cold PBS, harvested by scraping, pelleted and resuspended in cellular lysis buffer containing proteinase inhibitors (5 mM PIPES, 85 mM KCl, 0.5% NP-40, cOmplete protease inhibitor EDTA free (Roche)) and incubated 5 minutes on ice. After 5 minutes centrifugation at 600 g, the pellet was resuspended in 300 µl nuclear lysis buffer (50 mM Tris pH 8.0, 10 mM EDTA, 1% SDS, + protease inhibitor) and subjected to sonification using a Qsonica Q800R with constant chiller (40 minutes, 70% amplitude, 15 s on, 45 s off). Resulting fragment size typically was around 300 bp. Lysate was then incubated overnight with antibodies previously bound to Dynabeads Protein G (Invitrogen). Used antibodies were mouse-anti-FLAG M2 (catalog no. F3165, Sigma) or mouse-anti-GFP (clone 3E6, catalog no. A11120, Invitrogen). Complexes were washed twice with Dilution IP (16.7 mM Tris pH 8.0, 1.2 mM EDTA, 176 mM NaCl, 0.01% SDS, 1.1% Triton X-100), once with TSE buffer (20 mM Tris pH 8.0, 2 mM EDTA, 500 mM NaCl, 1% Triton X-100, 0.1% SDS), LiCl buffer (100 mM Tris pH 8.0, 500 mM LiCl, 1% deoxycholic acid, 1% NP-40), and finally twice with TE (10 mM Tris pH 8.0, 1 mM EDTA). Input control and beads were resuspended in elution buffer (50 mM NaHCO_3_, 140 mM NaCl, 1% SDS) and treated with RNase A and Proteinase K, before decrosslinking during 4 hours at 65 ˚C. MinElute columns (Qiagen) were used to extract DNA. qPCR analysis was carried out in a QuantStudio 3 (Thermo Fisher Scientific) with Luna Universal Probe qPCR Master Mix (NEB) and the Universal Probe system (Roche). Primers and probes used were: ADM2 promoter 5’-CGCCTGGATTCAAGCCTCA and 5’-GGGTGCCTGTAATCTCAGCT with probe #30, ADM2 exon 2 5’-GACACCCCTTTCTACCCCAG and 5’-AGGTGAGGTGGGTCTAGTCG with probe #23. Data was normalized to input controls.

### Luciferase reporter assays

HEK293T cells were cultivated at 37 °C and 5% CO_2_ in DMEM containing charcoal-stripped fetal bovine serum and penicillin-streptomycin (all Thermo Fisher). 10,000 cells per well were seeded into white 96-well plates with glass bottom (Greiner Bio-one) and transiently transfected with zebrafish Ar using Lipofectamine 3000 (Thermo Fisher). As plasmids we used such encoding the zebrafish Ar and reporter constructs with luciferase under the control of the *adm2a* promoter: A 1970 bp sequence upstream of the adm2a transcription start site and a short version of this sequence (1471 bp) were used to drive luciferase expression in the promoterless pGL4.10 (luc2) vector (Promega). As internal control for transfection efficiency, a plasmid encoding renilla luciferase (Promega) was co-transfected. A fragment of bacterial beta-galactosidase, which was similar in size as the *adm2a* fragments, was cloned into pGL4.10 (luc2) and used as negative control. Empty vector was used to allow for equal amounts of plasmid transfected in each well. One day after transfection, the medium was changed to culture medium containing either 100 nM dihydrotestosterone (DHT) (Sigma-Aldrich) or vehicle (methanol, final concentration: 0.1%). After 24 h of stimulation, firefly and renilla luciferase activity were measured using the Dual Glo Luciferase Assay system and a GloMax GM3000 plate-reader (both Promega). Readings were background subtracted using results from untransfected cells as suggested by the manufacturer’s manual and normalized to cells transfected with the zebrafish Ar without luciferase reporter constructs.

### Statistics

Data were analyzed using Prism 10 (GraphPad). First, data were tested for normal distribution using a Shapiro-Wilk normality test before the respective parametric or non-parametric test was applied. The α level was set to 0.05. Datasets with more than two conditions were analyzed with an ANOVA-based test. Please see figure legends for applied tests in specific experiments, numbers of experiments and embryos used, as well as represented measure of variability. Graphs generally represent either percentage of phenotypic embryos or individual embryos.

### Data Availability

The data underlying this article are available in the article and in its online supplementary material.

## Results

### Loss of Ar function impairs cardiac performance in zebrafish embryos

Recently, we performed a small chemical compound screen [15] to identify unanticipated or under-investigated candidate proteins controlling early vertebrate heart and specifically CCS development. One of the few compounds resulting in inconsistent pacing of the heart was PF-998,425, an inhibitor against the Ar. The Ar is a cytosolic receptor, which translocates into the nucleus upon binding to androgens such as androstenedione, testosterone and dihydrotestosterone. In the nucleus, the Ar functions as a transcription modulator altering the transcription of many target genes. Both, the receptor as well as the required enzymatic machinery to produce androgens are already expressed early during zebrafish development and long before the gonads develop [23] suggesting that the Ar likely impacts on embryonic development independently of gonad function. Hence, we generated a digoxigenin-labeled RNA probe based on a previous publication [23] and performed whole mount in situ hybridization. We detected *ar* transcripts as previously shown in the olfactory placode, but in addition also in the developing heart (Figure S1).

**Fig. 1:**
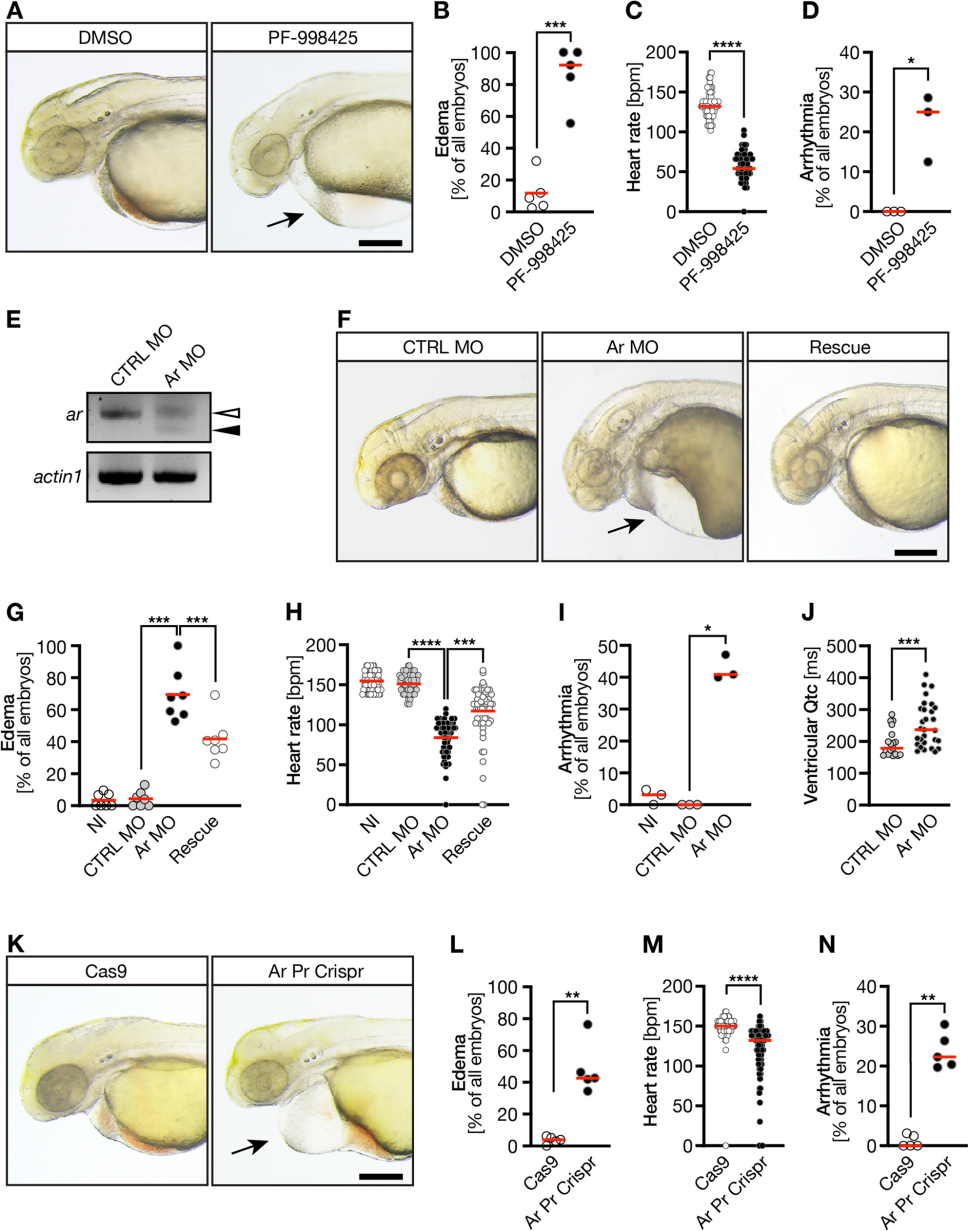
Loss of Ar function leads to impaired cardiac function during development. **A**, Live images of zebrafish embryos treated with vehicle (DMSO) or 50 µM PF-998,425. Arrow indicates edema in the pericardial cavity and before the inflow tract. **B**, Graph showing the percentage of embryos displaying edema in the heart region. *n*=5 experiments with 156–159 embryos in total. *** *p*=0.0001. Two-tailed Welch’s test. **C**, Ar blockade by PF-998,425 significantly decreases the heart rate in embryos. *n*= 3 experiments with 72–75 embryos in total. **** *p*<0.0001. Two-tailed Mann-Whitney test. **D**, PF-998,425 increases the number of arrhythmically beating hearts. *n*=3 experiments with 80–82 embryos in total. * *p*=0.0456. One sample t and Wilcoxon test. **E**, Zebrafish eggs were injected with a non-targeting control MO (CTRL MO) or a splice blocking MO targeting the pre-mRNA of the Ar (Ar MO). At 24 hpf, RT-PCR was performed to show splice blocking upon AR MO injection. White arrowhead: expected *ar* fragment size (size: 436 bp), black arrowhead: fragment lacking exon 3 (size: 319 bp). *Actin1* fragment size: 491 bp. Representative images of 2 experiments. **F**, Live images of injected zebrafish. Non-injected embryos (NI) served as clutch control. **G**, Graph summarizing the percentage of embryos with edema. *n*=7 experiments with 172–222 embryos in total. *** *p*=0.0003 (CTRL MO vs. Ar MO) and 0.0005 (Ar MO vs. Rescue). RM One-way ANOVA with Holm-Sidak’s multiple comparisons test. **H**, Bradycardia upon Ar splMO injection in embryos. *n*=3 experiments with 59–78 embryos in total. *** *p*=0.0001, **** *p*<0.0001. Kruskal-Wallis test with Dunn’s multiple comparison test. **I**, Arrhythmia can be also observed in Ar morphants. *n*=3 experiments with 76–81 embryos in total. * *p*=0.0102. Kruskal-Wallis test with Dunn’s multiple comparison posttest. **J**, Ar knockdown increases the Qtc. *n*=3 experiments with 19–29 embryos in total. *** *p*=0.0008. Two-tailed Mann-Whitney test. **K**, Live images of zebrafish embryos injected with Cas9 or Cas9 and three gRNAs against promoter of the *ar* (Ar Pr Crispr). Arrow indicates edema. **L**, Graph showing the percentage of embryos displaying edema. *n*=5 experiments with 177–189 embryos in total. * *p*=0.0033. Two-tailed Welch’s test. **M**, Ar Pr Crispr decreases the heart rate. *n*=5 experiments with 108–143 embryos in total. **** *p*<0.0001. Two-tailed Mann-Whitney test. **N**, Ar Pr crispants display arrhythmia more often than embryos injected with Cas9 only. *n*=4 experiments with 69–114 embryos in total. * *p*=0.0106. One sample t and Wilcoxon test. All data obtained at 48 hpf. Scale bars: 200 μm. Red line indicates median

**Fig. 2:**
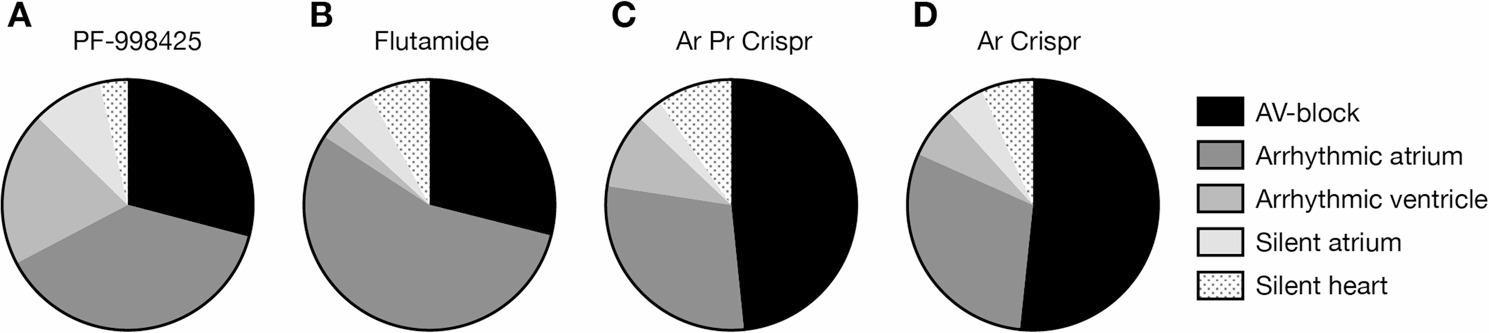
Arrhythmia types upon Ar LOF. **A**, Pie chart showing types of arrhythmia provoked by PF-998,425 treatment. *n*=6 experiments with 55 embryos in total. **B**, Types of arrhythmia after flutamide treatment. *n*=6 experiments with 38 embryos in total. **C**, Arrhythmia types in Ar promoter crispants. *n*=6 experiments with 60 embryos in total. **D**, Arrhythmia types in embryos injected with a ribonucleoprotein complex targeting exon 1. *n*=7 experiments with 31 embryos in total. Pie charts only depict arrhythmic embryos. Corresponding controls rarely showed arrhythmia

In order to analyze a potential Ar function during heart development, we applied several approaches to impair normal Ar function, namely pharmacological inhibition with three different AR blockers, knockdown and gene editing using Crispr/Cas9. First, we performed pharmacological inactivation by immersing the embryos in embryo water containing established Ar inhibitors. Embryos were always treated during organogenesis from tailbud stage on to avoid interfering with cell movements during gastrulation. Inactivation of Ar by treatment with the Ar inhibitor PF-998,425 from the tailbud stage on markedly affected cardiac performance with minor effects on the overall morphology of the embryo (Figure S2). We observed the development of edema in the pericardiac region and/or the inflow tract, pronounced bradycardia and arrhythmic beating, which included atrial arrhythmia and atrioventricular blocks (Fig. 1A-D). To ascertain that these effects were not mere off-targets or due to toxicity, we performed several control experiments. First, we tested by qPCR that known AR target genes were differentially expressed as described in the literature (Figure S3A and B). This confirmed blockade of the Ar. We also tested two additional, structurally distinct pharmacological inhibitors of the AR (Figure S2). Both, flutamide as well as ARN-509 caused edema formation and a reduced heart rate (Figures S2 and S4). As these inhibitors, however, were not developed for targeting the zebrafish Ar and may bind less efficiently to the fish receptor, we additionally employed a knockdown approach. Knockdown, in contrast to genetic deletion in stable mutant lines, prevents a regulatory feedback mechanism, which can mask potential phenotypes [33]. Injection of a splice blocking antisense morpholino oligonucleotide (Ar MO) interfered with regular splicing of the zebrafish *ar* RNA (Fig. 1E). This caused a very similar, if not even more pronounced phenotype as the inhibitory compounds. Subsequent co-injection of MO-insensitive, capped RNA encoding the Ar significantly rescued edema formation and increased the heart rate (Fig. 1F-H). Interestingly, this rescue was only partial which we attribute to Ar overexpression producing a mild bradycardia on its own (Figure S5). MO-mediated knockdown of Ar also led to arrhythmia as observed under pharmacological blockade (Fig. 1I). In addition to the rescue experiments and to assess the specificity of the MO, we analyzed Ar knockdown embryos for the same target genes as we did after pharmacological intervention and found regulation as seen after compound administration (Figure S3C and D). To corroborate our finding that Ar LOF causes beating irregularities, we measured the QTc. Embryos injected with the Ar MO had significantly longer QTc intervals than control injected embryos (Fig. 1J) consistent with the observed bradycardia. Last, but not least, we performed transient Crispr/Cas9 targeting the region upstream of the transcription start site of the Ar by simultaneously injecting three gRNAs (Figure S6 and S7) and compared the resulting embryos with those injected with Cas9 protein only. Ar promoter crispants displayed pericardiac and inflow tract edema, bradycardia and arrhythmia at 48 hpf (Fig. 1K-N), showed a reduction in Ar target gene expression (Figure S3E and F) and prolonged QTc intervals (Figure S8) as observed upon pharmacological Ar blockade and knockdown. In a second Crispr approach we targeted exon 1 of the *ar*, which resulted in a highly similar phenotype (Figure S9 and S10). Last, but not least, we assessed the arhythmic beating more closely. Loss of Ar function mostly resulted in arrhythmic atrial beating, which was characterized by irregular pauses or bursts of faster beating. Particularly in Ar LOF embryos with a slightly higher heart rate than the average Ar LOF embryo, different degrees (partial to complete) atrioventricular blocks could be observed. Small percentages of embryos also displayed arrhythmic beating of the ventricle, silent atria or even silent hearts (Fig. 2, Supplemental Movie 1). Taken together, these data suggest that transient loss of Ar function interferes with normal cardiac function during embryonic development.

### Ar LOF causes defects in cardiac morphology and in CCS patterning

**Fig. 3:**
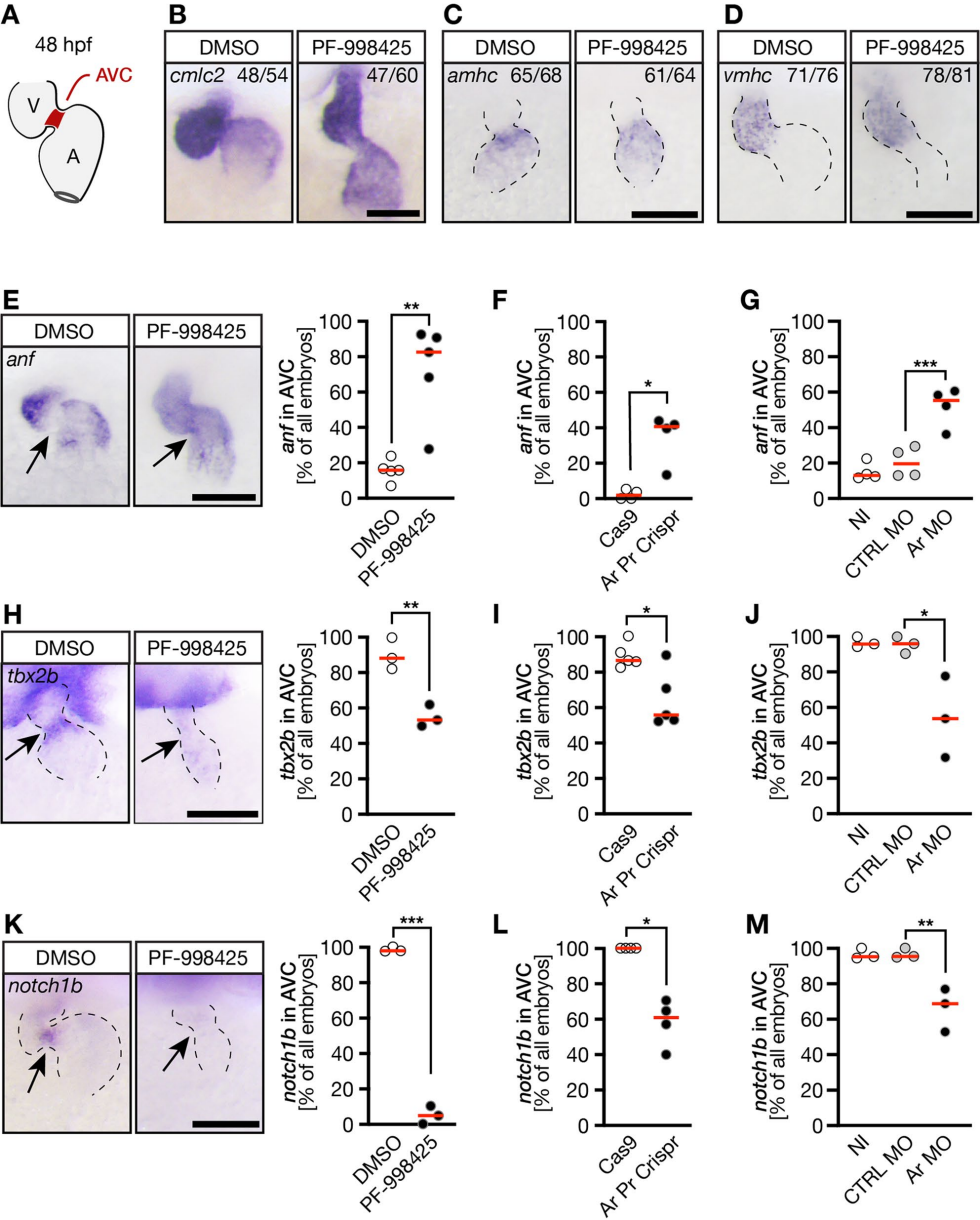
Ar is required for normal heart formation and AVC patterning. **A**, Cartoon showing compartments of the 48 hpf zebrafish heart. A, atrium; AVC, atrioventricular canal; V, ventricle. **B**, Ar blockade results in misshaped hearts. *n*=3 experiments. **C**, Atrium formation occurs normally in embryos with blocked Ar function. *n*=3 experiments. **D**, Ventricular specification is not affected by Ar inhibition. *n*=3 experiments. **E**, *Anf* is excluded from the AVC in vehicle-treated embryos, but expressed in the AVC in PF-998,425-treated embryos. *n*=5 experiments with 73–101 embryos in total. ** *p*=0.0078. Two-tailed Welch’s test. **F**, Ar promoter crispants display more often *anf* expression in the AVC than control embryos. *n*=4 experiments with 93–110 embryos in total. * *p*=0.0286. Two-tailed Mann-Whitney test. **G**, *Anf* is excluded from the AVC in control embryos, but expressed in the AVC in Ar morphants. *n*=4 experiments with 93–103 embryos in total. *** *p*=0.0006. One-way ANOVA with Holm-Sidak’s multiple comparison test. **H**, Atrioventricular *tbx2b* expression is lost upon pharmacological Ar blockade. *n*=3 experiments with 63–68 embryos in total. ** *p*=0.0071. Two-tailed Welch’s test. **I**, *Tbx2b* is less often enriched in the AVC in Ar promoter crispants. *n*=5 experiments with 86–94 embryos in total. * *p*=0.0210. Two-tailed Welch’s test. **J**, Atrioventricular *tbx2b* expression can be less often observed upon Ar knockdown. *n*=3 experiments with 66–74 embryos in total. * *p*=0.0103. One-way ANOVA with Holm-Sidak’s multiple comparison test. **K**, *Notch1b* transcripts fail to be enriched in the AVC in PF998425-treated embryos. *n*=3 experiments with 104–113 embryos in total. ***, *p*=0.0005. Two-tailed Welch’s test. **L**, Ar promoter crispants display less often *notch1b* enrichment in the AVC compared to Cas9 embryos. *n*=4 experiments with 63–68 embryos in total. *, *p*=0.0286. Two-tailed Mann-Whitney test. **M**, In contrast to control embryos, Ar depleted embryos fail to enrich *notch1b* in the AVC. *n*=3 experiments with 61–75 embryos in total. ** *p*0.0023. One-way ANOVA with Holm-Sidak’s multiple comparison test. All data WMISH at 48 hpf. All scale bars: 100 μm. Red line in all graphs indicates median. Arrows indicate AVC

To investigate whether morphological changes may underly the arrhythmia in embryos devoid of normal Ar function, we analyzed the developing zebrafish heart (Fig. 3A) in greater detail. Loss of Ar function by different means resulted in misshaped hearts (Fig. 3B). Nevertheless, chamber specification as assessed by the expression of the atrial and ventricular cardiac myosin genes was not affected (Fig. 3C and D). The abundance of chamber specific myosin RNAs, though, was lower at the level of cardiac progenitor cells which could potentially indicate a certain impairment in developing contractile elements (Figure S11). At least in the atrium, this did not prevent sarcomere development, as the sarcomeric structure of the atrium appeared normal in Ar promoter crispants. Furthermore, we did not see a reduction in the contractile gene *tnnt2* (Figure S12). Despite that, however, the ventricular ejection fraction was reduced in zebrafish embryos devoid of Ar function compared to controls (Figure S13).

Next, we analyzed the AVC upon Ar dysfunction. In situ hybridization for the *atrial natriuretic factor* (*anf)*, which is expressed in contractile myocardium during development and excluded from the non-contractile myocardium of the AVC, revealed *anf* expression in Ar LOF embryos throughout the entire heart including the AVC (Fig. 3E-G, Figure S14A and B). Similarly, *t-box transcription factor 2b* (*tbx2b*), which normally localizes to the myocardial part of the AVC [34, 35], as well as *notch1b*, which demarcates the endocardial CCS lineage of the AVC [36], failed to be enriched in the AVC in embryos devoid of normal Ar signaling (Fig. 3H-M, Figure S14C-F). Hence, we conclude that the Ar is required for normal patterning of the AVC and the conduction tissue within the AVC consistent with the atrioventricular blocks observed.

### Ar LOF impairs SAN function

**Fig. 4:**
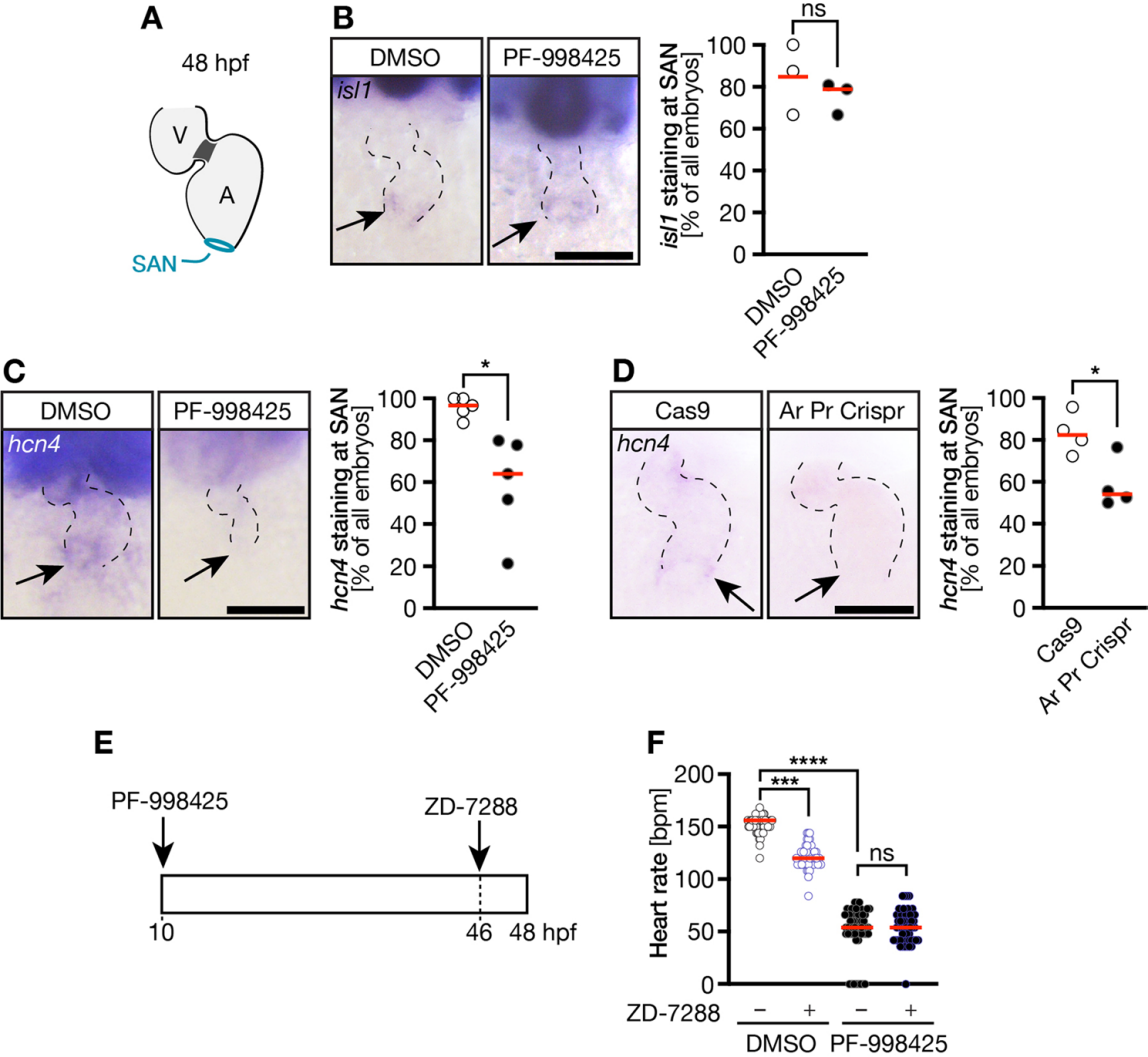
Ar LOF provokes impairs normal SAN development and function. **A**, Cartoon showing sinoatrial node (SAN) of the 48 hpf zebrafish heart. A, atrium; SAN, sinoatrial node; V, ventricle. **B**, *Isl1* expression as a marker for development of cells with a sinoatrial fate in DMSO- and PF-998,425-treated embryos. *n*=3 experiments with 61 embryos in total. ns, *p*=0.4553. Two-tailed Welch’s test. **C**, *Hcn4* expression is lost upon Ar blockade. *n*=5 experiments with 117–122 embryos in total. * *p*=0.0246. Two-tailed Welch’s test. **D**, Ar promoter crispants often fail to express *hcn4* in the region of the SAN. *n*=4 experiments with 117–122 embryos in total. * *p*=0.0211. Two-tailed Welch’s test. **E**, Treatment paradigm to test for Hcn channel functionality of the SAN. Embryos were treated with PF-998,425 (or vehicle) from 10 hpf on. Two hours before analysis at the 48 hpf stage, the HCN blocker ZD-7288 was added. **F**, ZD-7288 decreases the heart rate of control embryos, but is unable to do so in Ar LOF embryos. *n*=3 experiments with 47–54 embryos in total. **** *p*<0.0001. *** *p*=0.0004. Kruskal-Wallis test with Dunn’s multiple comparison test. All data WMISH at 48 hpf. All scale bars: 100 μm. Red line in all graphs indicates median. Arrows indicate SAN region

The most striking phenotype, however, was the bradycardia and the irregular beating on the level of the atrium. Therefore, we analyzed marker genes of the SAN (Fig. 4A), which we expected to show similar defects as the AVC. Expression of *islet1* (*isl1*), the key factor for the formation and functionality of the SAN, however, was not changed compared to controls, although zebrafish embryos lacking Isl1 display cardiac pacing defects very similar to those we observed upon Ar LOF [37]. Ar LOF embryos expressed *isl1* transcripts in the SAN region just the same as control embryos (Fig. 4B) suggesting that pacemaker cells per se do develop. As an indication whether these cells may still be functional, we analyzed the expression of *hyperpolarization activated cyclic nucleotide-gated potassium channel 4* (*hcn4*), a voltage gated ion channel. HCN4 is evolutionarily conserved and necessary for cardiac automaticity [38, 39]. As we found fewer embryos expressing *hcn4* in the SAN region upon administration of the Ar inhibitor PF-998,425 (Fig. 4C) and in Ar promoter crispants (Fig. 4D) we hypothesized that pacemaker cells still form in the absence of functional Ar, but the SAN as a tissue has possibly lost its full pacemaker function. Indeed, administration of ZD-7288, a general HCN channel blocker [40], reduced the heart rate in control embryos, but did not slow the heart of Ar LOF embryos further (Fig. 4E and F) suggesting that Hcn activity and with that SAN functionality cannot successfully be established in the absence of Ar function in zebrafish embryos.

### The Ar influences cardiac function by limiting *adrenomedullin 2a* (*adm2a*)

**Fig. 5:**
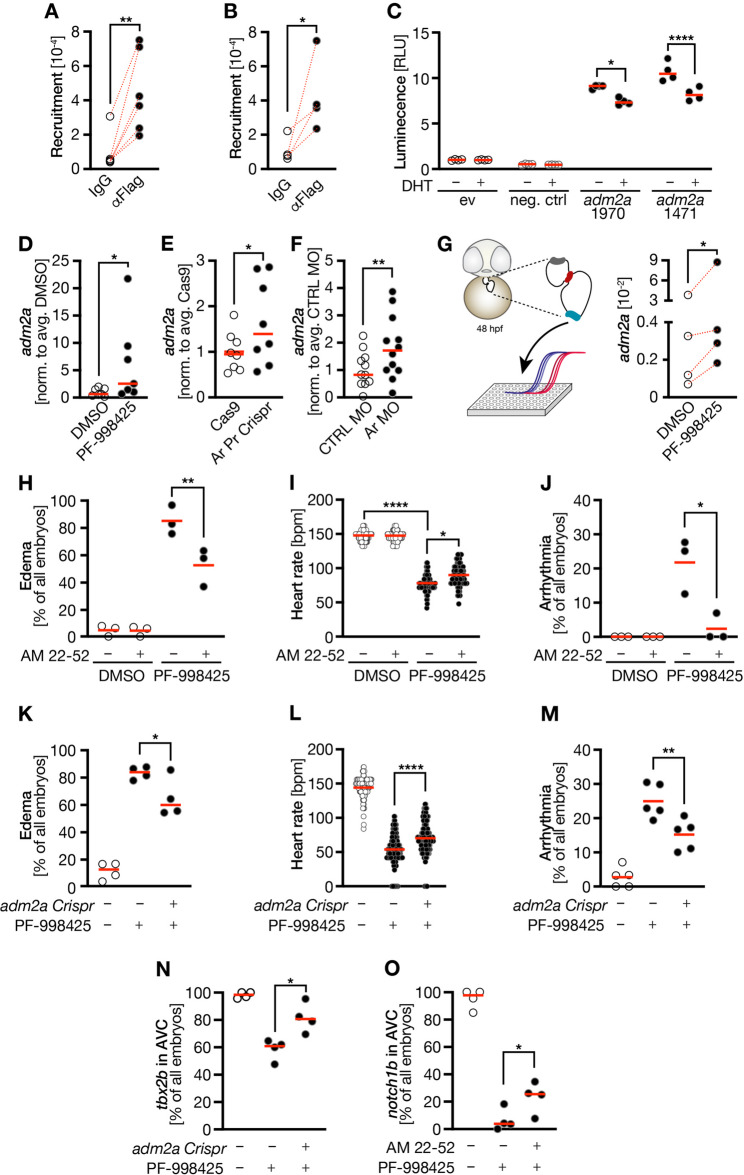
Ar LOF increases *adm2a* expression and ablation of Adm2a partially rescues heart function and AVC patterning. **A**, Chromatin immunoprecipitation in HEK293T cells transfected with Flag-Ar with control IgG or anti-Flag antibody reveals enrichment of a DNA fragment in exon 2 of human *ADM2*. Shown are results normalized to the input fraction. *n*=6. ** *p*=0.0087. Two-tailed Mann-Whitney test. **B**, Chromatin immunoprecipitation in HEK293T cells transfected with Flag-Ar with control IgG or anti-Flag antibody showing enrichment of a DNA fragment upstream of the transcription start site of human *ADM2*. Shown are results normalized to the input fraction. *n*=4. ** *p*=0.0286 Two-tailed Mann-Whitney test. **C**, Luciferase reporter assay in HEK293T cells transiently transfected with zebrafish Ar and either empty control vector (ev), a negative control (neg. ctrl) or luciferase under the control of sequences upstream of the transcription start site of zebrafish *adm2a*. Shown are basal levels and such stimulated with dihydrotestosterone (DHT). *n*=4. **** *p*<0.0001, * *p*=0.0358. One-way ANOVA with Sidak’s multiple comparison test. **D**, qPCR showing increased expression of *adm2a* in PF-998,425-treated embryos. *n*=7. * *p*=0.0156. Two-tailed Wilcoxon matched-pairs signed rank test. **E**, Ar promoter crispants have increased *adm2a* expression compared to Cas9-injected embryos. *n*=8. * *p*=0.0493. Two-tailed paired t-test. **F**, *Adm2a* transcripts are elevated upon Ar knockdown. *n*=12. ** *p*=0.0071. Two-tailed paired t-test. **G**, qPCR of isolated hearts demonstrates *adm2a* upregulation in PF-998,425-treated embryos. Graph shows absolute expression normalized to *gapdh*. *n*=4 experiments. * *p*=0.0410. Two-tailed ratio paired t-test. **H**, Administration of the ADM2 inhibitor AM 22–52 partially rescues edema formation in embryos treated with an Ar blocker. *n*=3 experiments with 80–86 embryos in total. ** *p*=0.0025. One-way ANOVA with Holm-Sidak’s multiple comparison test. **I**, AM 22–52 significantly increases the heart rate of Ar LOF embryos. *n*=3 experiments with 80–84 embryos in total. * *p*=0.0169, **** *p*<0.0001. Kruskal-Wallis test with Dunn’s multiple comparison test. **J**, AM 22–52 rescues arrhythmia induced by Ar blockade. *n*=3 experiments with 80–84 embryos in total. * *p*=0.0433. Kruskal-Wallis test with Dunn’s multiple comparison posttest. **K**, Adm2a depletion using transient Crispr/Cas9 reduces the percentage of Ar LOF embryos developing edema. *n*=4 experiments with 80–84 embryos in total. * *p*=0.0255. One-way ANOVA with Holm-Sidak’s multiple comparison test. **L**, Adm2a abrogation by Crispr/Cas9 significantly increases the heart rate of Ar blocker-treated embryos. *n*=5 experiments with 159–177 embryos in total. **** *p*<0.0001. Kruskal-Wallis test with Dunn’s multiple comparison test. **M**, Crispr/Cas9 against *adm2a* significantly rescues arrhythmia in embryos undergoing Ar blockade. *n*=5 experiments with 159–173 embryos in total. ** *p*=0.0031. One-way ANOVA with Holm-Sidak’s multiple comparison test. **N**, gRNA injection against *adm2a* results in more Ar LOF embryos expressing *tbx2b* in the AVC compared to Ar blockade alone. *n*=4 experiments with 81–107 embryos in total. * *p*=0.01701. Brown-Forsythe and Welch ANOVA test with Dunnett’s T3 multiple comparison test. **O**, Administration of AM22-52 increases the percentage of embryos with *notch1b* enrichment in the AVC. *n*=4 experiments with 80–97 embryos in total. * *p*=0.0261. One-way ANOVA test with Holm-Šídák’s multiple comparisons test. All data obtained at 48 hpf. Red line: median. Red dotted lines indicate experimental pairs

Cellular signaling events controlled by the Ar and ways to ameliorate the consequences thereof have been extensively analyzed in the context of prostate cancer [41, 42]. Using this resource we looked for Ar target genes with known cardioactivity, which might also be regulated in the developing heart. We identified Adm as a candidate, which is increased under conditions of AR inhibition [43]. Adm is expressed in the developing mouse heart [44] and requires tight regulation to allow for normal cardiogenesis as elevated expression of Adm or its receptor results in congenital heart defects in mice [45, 46]. In P19 cells, which we differentiated towards cardiac cells (Figure S15A and B), we observed a tendency towards higher Adm expression, when Ar was depleted (Figure S15C). We hence performed chromatin immune precipitation experiments in HEK293T cells transiently transfected with Flag-tagged zebrafish Ar (Figure S16A and B) and performed qPCR for exon 2 of human ADM, which appears to contain a cryptic androgen responsive element and for the promoter of ADM2. In cells stimulated for 3 h with 1 nM dihydrotestosterone (DHT), we found translocation of the receptor to the nucleus (Figure S15B) and recruitment of the Ar to both sequences in DHT-stimulated cells (Fig. 5A and B). To see whether zebrafish Adm2a, a close homolog of mammalian ADM2, also represents a target of the Ar, we performed reporter assays in HEK293T cells transfected with zebrafish Ar using a larger (1970 bp) and a smaller (1471 bp) fragment of the presumptive promoter of *adm2a* to drive luciferase expression. While empty vector and a control sequence comprised by a fragment of bacterial beta-galactosidase did not strongly increase luminescence, we observed robustly elevated signals with the sequences upstream of the *adm2a* transcription start site suggesting that *adm2a* may be a target gene of the Ar in zebrafish. Interestingly, we observed luciferase activity already in unstimulated cells and detected slightly, but significantly lower signals upon stimulation with dihydrotestosterone (Fig. 5C), which may be explained by low nuclear Ar localization already under unstimulated conditions (Figure S15C).

Next, we assessed *adm2a* expression in zebrafish embryos. Pharmacological blockade of Ar, Ar loss by gene-editing and knockdown resulted in increased abundance *adm2a* in whole embryos (Fig. 5D-F, Figure S17A). Furthermore, qPCR analysis of isolated hearts revealed *adm2a* upregulation upon PF-998,425 treatment (Fig. 5G), which we confirmed by in situ hybridization (Figure S17B). In contrast, treatment with the muscarinic receptor blocker tolterodine, which also causes arrhythmia in zebrafish embryos [15], did not produce an increase in *adm2a* expression (Figure S18).

We hence hypothesized that reducing the Adm2a load in Ar LOF embryos may ameliorate the observed heart phenotypes. We employed a two-fold strategy using on the one hand a small peptide inhibitor of human Adm on embryos treated simultaneously with the Ar blocker, while on the other hand we reduced Adm2a expression with a transient Crispr/Cas9 approach (Figure S19). Both strategies decreased the percentage of embryos displaying edema formation in the vicinity of the heart indicating an improvement of heart function. This was confirmed by heart rate analysis as both approaches increased the heart rate of Ar blocker-treated embryos to a small, but nevertheless significant extend. In addition, the fraction of embryos showing arrhythmia of any kind was reduced (Fig. 5H-M), which was consistent with an increased percentage of embryos correctly expressing *tbx2b* and *notch1b* in the AVC (Fig. 5N and O). These data suggest that at least parts of the cardiac phenotype provoked by Ar LOF may be due to unleashed *adm2a* expression. Possibly, this function of Adm2a is mediated via the Adm receptor 1 as we detected *receptor activity modifying* protein 2 (*ramp2)*, the essential component of this receptor expressed in the region of the presumptive proepicardium (Figure S20). In summary, these data suggest a direct regulation of *adm2a* by the Ar, which appears to contribute to the development of the observed Ar loss-of-function phenotypes. Further experiments such as recapitulating the phenotypes by Adm2a overexpression or assessing transcriptional regulation of *adm2a* directly in cells of the developing heart will be instrumental in strengthening the results obtained in heterologous cell systems.

### Proepicardium development is compromised upon Ar LOF

**Fig. 6:**
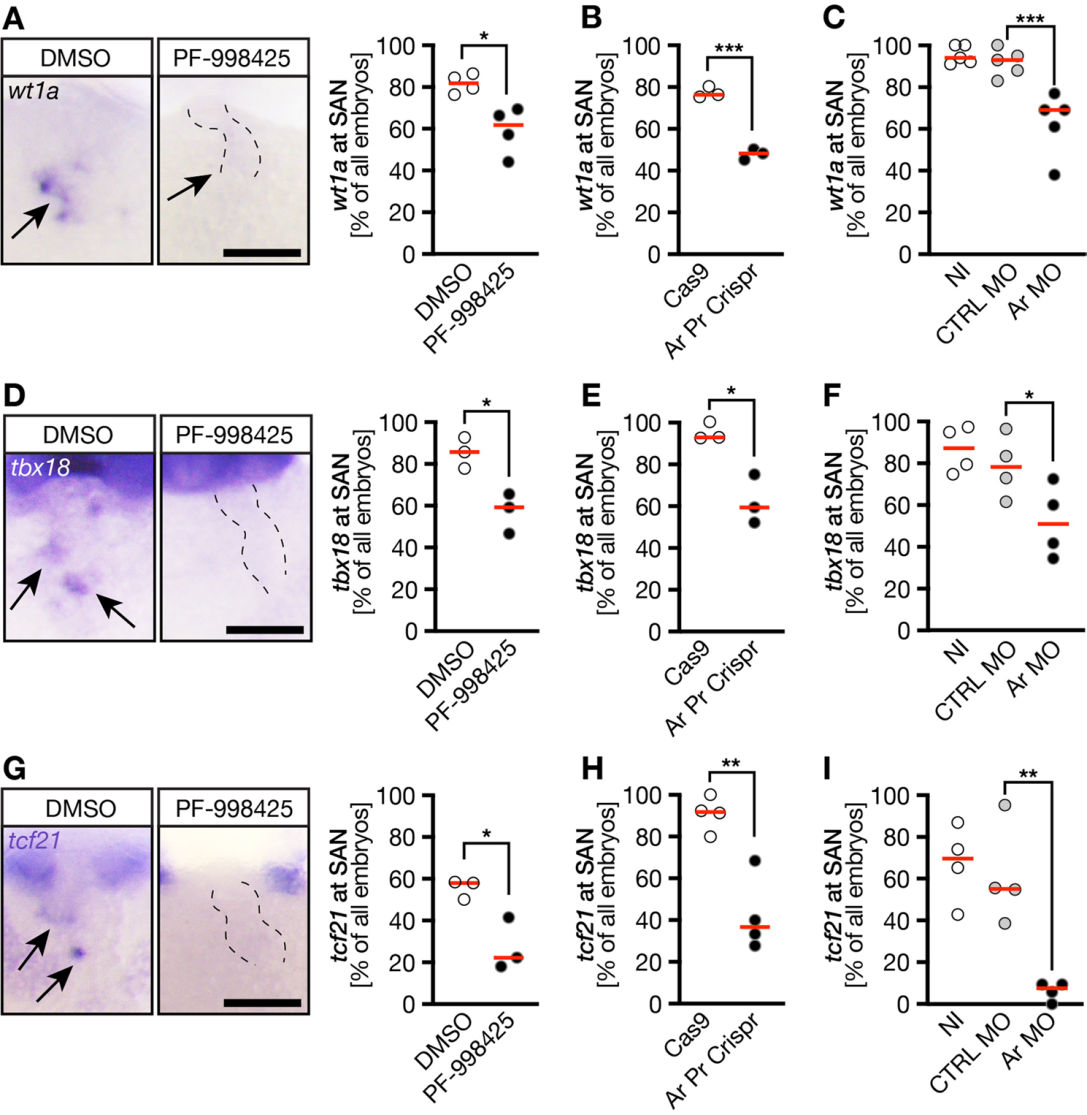
Proepicardium formation depends on Ar function. **A**, When Ar function has been blocked, the proepicardium marker *wt1a* is less often expressed in the SAN region. *n*=4 experiments with 112–116 embryos in total. * *p*=0.0217. Two-tailed Welch’s test. **B**, Ar Pr crispants display less often *wt1a* in the area of the SAN. *n*=3 experiments with 61–69 embryos in total. *** *p*=0.0002. Two-tailed Welch’s test. **C**, Ar morphants express less often *wt1a* in the vicinity of the SAN. *n*=5 experiments with 105–138 embryos in total. *** *p*=0.0003. One-way ANOVA with Sidak’s multiple comparison test. **D**, Upon Ar blockade, fewer embryos display *tbx18* expression at the SAN. *n*=3 experiments with 82–89 embryos in total. * *p*=0.0181. Two-tailed Welch’s test. **E**, *Tbx18* expression at the SAN can be less often seen in Ar Pr Crispr embryos. *n*=3 experiments with 59–72 embryos in total. * *p*=0.0283. Two-tailed Welch’s test. **F**, Ar knockdown results in fewer embryos with *tbx18* at the SAN. *n*=4 experiments with 136–151 embryos in total. * *p*=0.0312. One-way ANOVA with Sidak’s multiple comparison test. **G**, PF-998,425 treatment reduces the percentage of embryos expression *tcf21* in the region of the SAN. *n*=3 experiments with 48–90 embryos in total. * *p*=0.0459. Two-tailed Welch’s test. **H**, Tcf21 can be less often observed in the area of the SAN in Ar promoter crispants than in control-injected embryos. *n*=4 experiments with 58–64 embryos in total. ** *p*=0.0072. Two-tailed Welch’s test. **I**, *Tcf21* is lost upon Ar knockdown. *n*=4 experiments with 68–108 embryos in total. ** *p*=0.0018. One-way ANOVA with Holm-Sidak’s multiple comparison test. All data obtained at 48 hpf. Scale bars: 100 μm

In mouse embryos, Adm-mediated control of normal heart development originates from the proepicardium [45]. The proepicardium is an intermediate tissue in the dorsal pericardium, located behind the atrium and in many cases close to the inflow tract or the AVC [32, 47, 48]. It provides essential progenitor cells not only for the later epicardium, but also for many other cell types and compartments in the mature heart [49, 50]. Most importantly, the epicardium derived from the proepicardium has been shown to be instrumental for the regular pacemaking activity of the SAN [18, 51] and deficiencies in epicardial function appear to contribute to the development of ventricular as well as atrial arrhythmias in patients [51]. The proepicardium consists of a heterogeneity of cells which can be detected by the expression of Wt1a, Tbx18 and Tcf21 [52]. We tested two of these genes in our P19 differentiation model and detected reduced proepicardial gene expression in Ar knockdown cells (Figure S15D and E). Similarly, *wt1a*, *tbx18* and *tcf21* were less often expressed in the SAN region of zebrafish embryos depleted of Ar function at 48 hpf (Fig. 6A-I). At a later stage, we observed a deficiency of the proepicardium spreading over the developing heart (Figure S21). Moreover, the WT1 interacting protein (Wtip), which reportedly functions upstream of Tbx18 and Tc21 [53], was also reduced upon Ar knockdown (Figure S22a). Taken together, these data suggest a yet unanticipated role for the Ar in proepicardium patterning and potentially development.

### Bmp2b-mediated proepicardium induction partially rescues AR LOF phenotypes

**Fig. 7:**
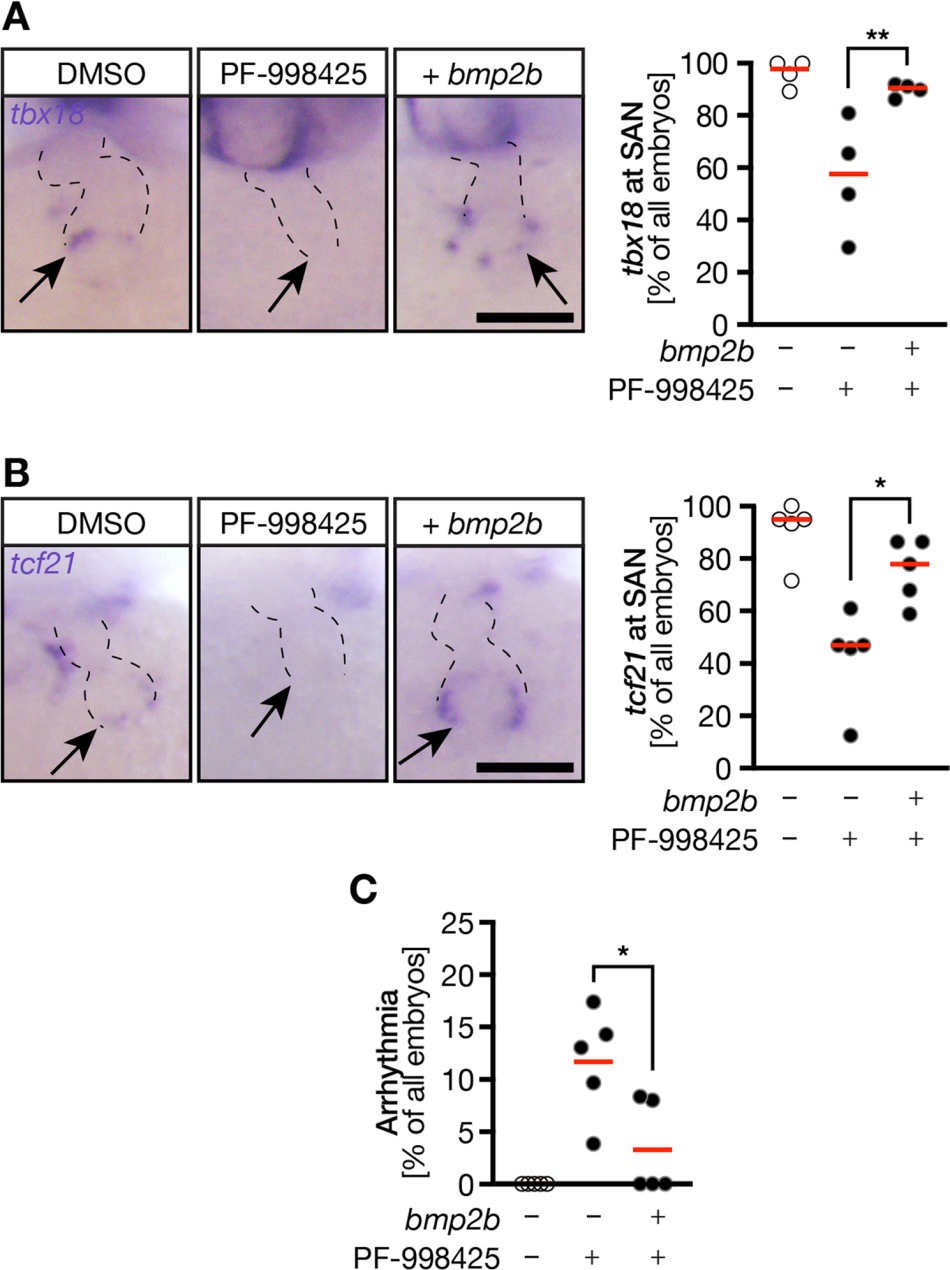
Supplementation with Bmp2b partially rescues proepicardium formation. **A**, *Tbx18* expression at the SAN is restored by Bmp2b overexpression. *n* = 4 experiments with 120–125 embryos in total. ** *p* = 0.0056. One-way ANOVA with Holm-Sidak’s multiple comparison test. **B**, Proepicardial *tcf21* expression is rescued by *bmp2b* RNA injection. *n* = 5 experiments with 112–126 embryos in total. * *p* = 0.0114. One-way ANOVA with Holm-Sidak’s multiple comparison test. **C**, Bmp2b overexpression rescues arrhythmia in Ar LOF embryos. *n* = 5 experiments with 123–137 embryos in total. * *p* = 0.0288. Kruskal-Wallis test with Dunn’s multiple comparison test. All data obtained at 48 hpf. Scale bars: 100 μm

In order to scrutinize our hypothesis that the Ar allows for normal SAN function by enabling proepicardium development, we attempted to enforce proepicardium formation through Bmp2b overexpression. Previously, this approach has been successfully applied to induce the expression of proepicardial genes in zebrafish [47, 54]. We injected embryos at the 1–2 cell stage with capped RNA encoding zebrafish Bmp2b producing only low Bmp2b expression to avoid severe ventralization and blocked Ar function using PF-998,425 from the tailbud stage on. This approach significantly rescued proepicardial marker gene expression suggesting that proepicardium development was at least partially restored in Ar-blocked embryos (Fig. 7A and B). Also, functionally, we could partly rescue the Ar LOF phenotype using Bmp2b overexpression. We observed a trend towards fewer edema, normalized Qtc, and improved ejection fraction (Figure S23A-C). Most importantly, the percentage of embryos displaying arrhythmia was again similar to control embryos (Fig. 7C). Unfortunately, however, the heart rate could not be rescued (Figure S23D), which may be possibly explained by Bmp2b having an inhibitory effect on ventricular contractility [55]. These data suggest a novel role of the Ar in regulating heart development and function through Adm2a and the proepicardium. The data further suggests the potential involvement of additional signaling pathways as of the rescue experiments yielding significant, but partial effects.

## Discussion

In the aging adult, impaired Ar signaling or insensitivity to androgens is considered a predisposing factor for cardiovascular disease including arrhythmia [56]. The outcome of androgen-mediated signaling on the developing heart, however, has not attracted much attention although mice lacking the Ar develop smaller hearts [14] and although the Ar is already expressed in the developing vertebrate heart [12, 13]. Perhaps, androgen function before gonad development and massive testosterone production simply has not been considered previously. Moreover, studies using stable, genetic Ar zebrafish did not include or report any cardiovascular analyses [57–59]. Such a non-existing or yet unreported cardiac phenotype arising from Ar deficiency in zebrafish may be due to protective, regulatory feedback mechanisms, which have the potential to mask functions of a given gene, when permanently deleted [33]. In the case of androgens, such regulatory mechanisms are likely as there is more than one Ar, namely one canonical nuclear receptor and one non-canonical, non-nuclear receptor named Gprc6a. A recent study in zebrafish, which used transient Crispr/Cas9 to delete Gprc6a, nicely demonstrated that this non-nuclear Ar mediates some of the cardiac effects, namely the pericardiac edema, evoked by androgen administration [8]. Hence, heart defects in cases of permanent loss of the canonical Ar may be obscured or at least substantially reduced due to Gprc6a skipping in. Consistent with such hypothesis may be individuals with complete or partial androgen insensitivity syndrome (CAIS or PAIS), a form of insufficient sexual maturation due to LOF mutations in the canonical, nuclear Ar. CAIS and PAIS patients do not generally present with CHDs, but are more prone to cardiovascular disease as adults. In contrast, individuals with sex chromosome aberrations have a high risk of CHDs, which considerably exceeds the prevalence of CHDs in the normal population [5]. Indeed, a large number of Klinefelter (47,XYY) as well as Turner syndrome (45X,0) patients suffer from malformations of the heart [60–62]. Interestingly, in Klinefelter and Turner syndrome testosterone levels are drastically below normal [6, 7], which could possibly reduce activation of both, canonical as well as non-canonical Ar. In consequence, signaling may fall below a threshold for normal receptor function, while still being high enough to prevent induction of further regulatory processes. CAIS and PAIS patients, on the other hand, have normal or slightly elevated testosterone levels [63], which could still activate GPRC6A. Hence, we speculate that the experimental conditions in our study might reflect Ar signaling levels in Klinefelter and Turner syndrome. In line with this, in several patients with these syndromes, arrhythmia has been observed (i.e. sinus bradycardia, sick sinus syndrome) [64–67], as well as QTc elongation [62] and ventricular dysfunction [61], which reflects our observations in zebrafish. We thus propose that the majority of cardiac phenotypes reported in here are due to inactivation of the nuclear Ar, not least because effects seen upon pharmacological inhibition were phenocopied using a knockdown and two independent Crispr strategies, which do not target Gprc6a. Further experiments could help to understand whether and if so, to which extent pharmacological Ar inhibitors would also block Gprc6a signaling in zebrafish. For human GPRC6A, however, we have not found any evidence that the pharmacological inhibitors would also bind to GPRC6A and modulate its function.

While the myogenic potential of nuclear Ar signaling has been published [11], much less is known about a potential impact on the developing CCS. Our results suggest that the Ar not only is involved in tissue patterning within the heart, but also promotes differential expression and functionality of cardiac ion channels such as Hcn4. Interestingly, certain ion channels required for cardiac pacing are differentially expressed in Ar-deprivation therapy experiencing patients [11, 68, 69].

Our data moreover suggests that the Ar possibly also regulates a proepicardial cell fate, which could contribute to the observed defects in cardiac rhythmicity. The proepicardium is located in the direct vicinity of the AVC and needs to intermingle with SAN cells for full pacemaking functionality [18, 32, 48, 50, 70]. Loss of *wt1* reduces the size of the SAN in mice and decreases the expression of both, SAN genes (i.e. *shox2*) as well as proepicardial genes (i.e. *tcf21*). Functionally, this results in arrhythmia and longer QRS intervals [71], similar to Ar LOF in zebrafish. Consistent with case reports in patients, we obtained data implicating Adm2a as a possible link between the Ar, the proepicardium and the functionality of the SAN as well as the AVC. In patients, Adm has been associated with arrhythmia [72, 73] and heart defects in mouse embryos could be evoked by proepicardium-specific overexpression of Adm. In our hands, interference with Adm2a function either by Crispr/Cas9 or by an inhibitor ameliorated not only the arrhythmic beating, but improved also the patterning of the AVC. Further experiments directly in cells of the developing heart as well as Adm2a overexpression, preferentially directly in the proepicardium, could help to further corroborate this hypothesis. Even more, as the rescues obtained were only partial, which may be explained by a possible involvement of Gprc6a, additional Adm proteins or parallel signaling pathways linked to Ar function. In summary, we propose a new mechanism by which the Ar regulates heart development and function before a biological sex has been determined. Based on our observation of reduction of proepicardial markers together with amelioration of arrhythmia by Bmp2b-mediated induction of the proepicardium (despite partial or insufficient rescue of edema and heart rate) we propose that fully functional Ar is required for faithful development and performance of the heart possibly partially through an adrenomedullin-proepicardium axis.

## Conclusions

Taken together, we identified a novel role of the Ar during heart development and propose impaired nuclear Ar signaling as one potential mechanism contributing to the high prevalence of CHDs in individuals with sex chromosome abnormalities.

### Limitations of the study

This study relies on a variety of transient approaches to achieve loss of Ar function and is hence not appropriate for drawing conclusions regarding electrophysiological outcomes or long-term effects of embryonic Ar dysfunction on the mature CCS. Functional electrophysiological measurements have not been conducted, which could specifically illuminate CCS function. We would like to emphasize that the presented rescue experiments corrected the assessed phenotypes significantly, although only partially, which suggests that additional pathways relevant for the embryonic heart may be affected by Ar LOF and contribute to the phenotypes. This study also does not address an extracardiac or general impact of the Ar on embryonic development. All LOF approaches used resulted in embryos with smaller heads, which is consistent with previous studies reporting increased proliferation of neural progenitors in the presence of testosterone [74] and the sex-related difference in brain size with males developing a larger brain mass. There are no good cell culture or animal models for Klinefelter or Turner syndrome, which could confirm or reject the hypotheses developed in this study.

## Supplementary Information

Supplementary Material 1.

Supplementary Material 2.

Supplementary Material 3.

Supplementary Material 4.

## Data Availability

No datasets were generated or analysed during the current study.

## Abbreviations

CHD: Congenital heart defect
AR: Androgen receptor
Adm: Adrenomedullin
Gprc6a: G protein-coupled receptor, class C, group 6, member A
CCS: Cardiac conduction system
DHT: Dihydrotestosterone
SAN: Sinoatrial node
AVC: Atrioventricular canal
Bmp2b: Bone morphogenetic protein 2b
ramp2: Receptor activity modifying protein 2
tcf21: Transcription factor 21
wt1a: Wilms tumor 1a
LOF: Loss-of-function
MO: Morpholino oligonucleotide
gRNA: Guide RNA
hpf: Hours post fertilization
WMISH: Whole mount in situ hybridization
tbx8: T-box transcriptiona factor 8
qPCR: Quantitative polymerase chain reaction
anf: Atrial natriuretic factor
isl1: Islet 1
hcn4: Hyperpolarization activated cyclic nucleotide-gated potassium channel 4
ramp2: Receptor activity modifying protein 2
Wtip: WT1 interacting protein
CAIS: Complete androgen insensitivity syndrome
PAIS: Partial androgen insensitivity syndrome
