# Supplementary material for: Cardiac function in zebrafish embryos is linked to an androgen receptor-adrenomedullin-proepicardium axis
Source: Cell Commun Signal. 2026 Jul 30;24:425. doi: 10.1186/s12964-026-03107-4 (PMC13422332; doi:10.1186/s12964-026-03107-4)
Supplement: Supplementary file 1 — Supplementary Material 1. [file 12964_2026_3107_MOESM1_ESM.pdf]

Duong Phu et al, Supplement

Table S1: qPCR primer sequences und Universal probe numbers.

Figure S1: Expression of androgen receptor (Ar) in the developing heart

Figure S2: Pharmacological Ar inactivation from tailbud stage on has minor effects on gross morphology of the embryos.

Figure S3: Target genes of the human AR are less expressed upon Ar LOF.

Figure S4: The Ar inhibitors flutamide and ARN-509 impair cardiac function similarly to PF-998425.

Figure S5: Ar overexpression produces bradycardia.

Figure S6: Gene editing efficiency of guide RNAs targeting the presumptive promoter of *ar*.

Figure S7: Overall morphology of Ar promoter crispants.

Figure S8: Qtc of Ar promoter crispants

Figure S9: Gene editing efficiency of *ar* gRNAs targeting exon 1.

Figure S10: Ar exon crispants display impaired cardiac function

Figure S11: Ar depletion causes a reduction in the size of the cardiac progenitor heart fields.

Figure S12: Sarcomere structure and contractile gene expression is not altered in Ar LOF embryos.

Figure S13: Ejection fraction analysis of embryos upon PF-998425 administration and gene-editing using Crispr/Cas9.

Figure S14: Representative images of in situ hybridizations for AVC marker genes.

Figure S15: Gene regulation during cardiogenic differentiation of P19 cells.

Figure S16: Transient transfection of zebrafish Ar into HEK293T cells.

Figure S17: Upregulation of *adm2a* expression.

Figure S18: The arrhythmogenic compound tolterodine does not induce increased *adm2a* expression.

Figure S19: Gene editing efficiency of *adm2a* gRNAs.

Figure S20: *Receptor (calcitonin) activity modifying protein 2* (*ramp2*) expression at 48 hpf.

Figure S21: Tcf21 expression at 72 hpf.

Figure S22: *Wtip* is reduced upon Ar knockdown.

Figure S23: Effects of Bmp2b overexpression.

Supplementary references

Table S1: qPCR primer sequences und Universal probe numbers.

organism	gene	forward	reverse	detection
d.r.	*adm2a*	TTT CCC GGT TTT TGT GTA TTG	GGA GTC TTG TCA TGA AGT CCA A	UP 108
d.r.	*adra1b*	TGG TTG GCA TGT TTA CGC	CCG AAG ACT GGT GTT GAA TG	UP 71
d.r.	*ar*	CGG AAG CTG CAA AGT GTT CT	TCC TGC TAG CGC ACA GAT AC	UP 18
d.r.	*igf1*	TTC AAA CAA GTT CAT TTT TGC TG	GAG ACA GCG CAT GGT ACA CTT	UP 67
d.r.	*tnnt2a*	CGA CGC AAA CCT CTG GAC AT	CGC ATC CAG CTC CAC AAT TC	UP 108
d.r.	*wtip*	CTA ACT GCG TGG TCA GTC CC	CAT GGA TCC CCT GGA GCT TC	UP 5
d.r.	*gapdh*	CAG GCA TAA TGG TTA AAG TTG GTA	CAT GTA ATC AAG GTC AAT GAA TGG	UP 147
m.m.	*GATA4*	AAA ACG GAA GCC CAA GAA CCT	TGC TAG TGG CAT TGC TGG AGT	SYBR
m.m.	*ACTC1*	CTG GTA TTG CCG ATC GTA TG	CTT GCT GAT CCA CAT TTG CT	SYBR
m.m.	*ADM1*	GCA GGG CCA GAT ACT CCT TC	TCT CAT CAG CGA GTC CCG TA	SYBR
m.m.	*TCF21*	GGC CAA CGA CAA GTA CGA GA	GTT TGC CGG CCA CCA TAA AG	SYBR
m.m.	*TBX18*	GGA TAT TGT GCC GGT GGA CA	CCG GGG AAT CAG CAT TTC CT	SYBR
m.m.	*GAPDH*	GGG TCC CAG CTT AGG TTC ATC	TAC GGC CAA ATC CGT TCA CA	SYBR

Sequences of primers used in qPCR experiments are given in 5'-3' orientation. Organism (d.r., danio rerio; mus musculus, m.m.) and analyzed genes are indicated. Amplification was detected either via Universal Probe (UP, Roche) or SYBR green. Numbers of used UP probes are indicated in last column.

SUPPLEMENTARY FIGURES

Figure S1: Expression of androgen receptor (Ar) in the developing heart

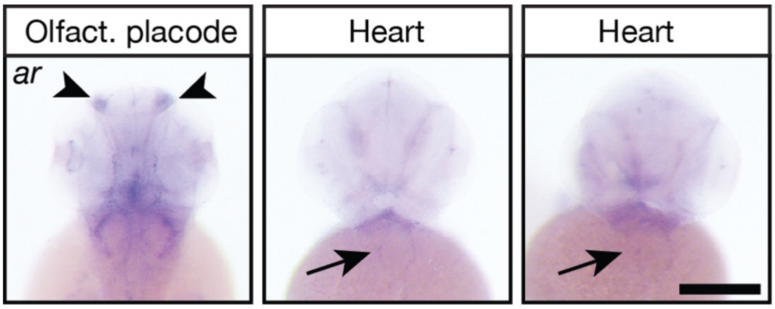

Ar expression could be detected in tissues such as the olfactory placode (arrowhead), but also in the developing heart (arrows).
Whole mount *in situ* hybridization at 48 hours post fertilization (hpf).
Scale bar: 200 µm.

Figure S2: Pharmacological Ar inactivation from tailbud stage on has minor effects on gross morphology of the embryos.

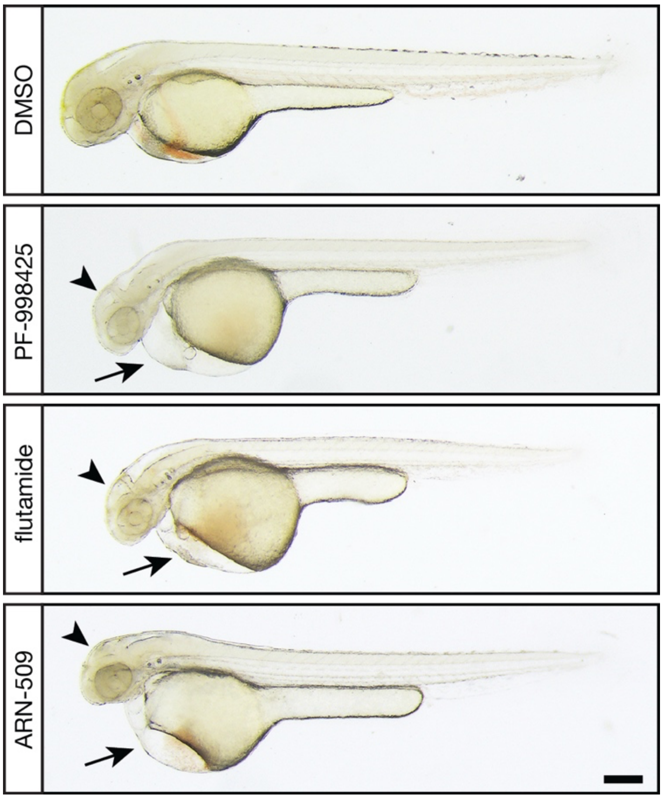

Live images at 48 hpf. Embryos were treated from tailbud stage until 48 hpf.
Arrows indicate edema in the pericardiac cavity and the inflow tract. Arrowheads indicate smaller heads.
Scale bar: 200 µm.

Figure S3: Target genes of the human AR are downregulated upon Ar LOF.

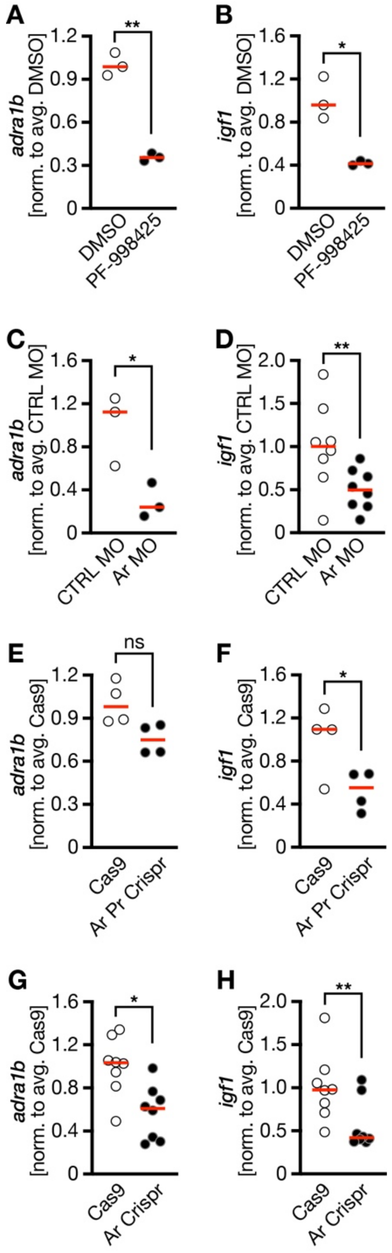

Zebrafish embryos were manipulated as indicated in the graphs. At 24 hpf, total RNA was extracted from a pool of embryos and qPCR was performed in triplicates for genes known to be positively regulated by androgen signaling in humans [1, 2].

A, Graph showing *adra1b* downregulation by PF-998425. n = 3. ** p = 0.0034. Two-tailed paired t-test.
B, Igf1 is decreased upon Ar blockade. n = 3. * p = 0.0309. Two-tailed paired t-test.
C, Ar knockdown reduces *adra1b*, too. n = 3. * p = 0.0466. Two-tailed paired t-test.
D, *Igf1* is significantly down after Ar MO injection. n = 8. ** p = 0.0060. Two-tailed paired t-test.
E, A*dra1b* expression in embryos injected with Cas9 or Cas9 and guide RNAs targeted against the promoter of *ar*. n = 4. ns, p = 0.0810. Two-tailed paired t-test.
F, *Igf1* is significantly down in Ar promoter crispants. n = 4. * p = 0.0170. Two-tailed paired t-test.

G, Compared to Cas9 injection alone, *adra1b* expression is reduced in embryos injected with guide RNAs targeted against *ar.* n = 8. ** p = 0.0460. Two-tailed paired t-test.
H, *Igf1* is significantly down in Ar crispants. n = 8. ** p = 0.0076. Two-tailed Wilcoxon matched-pairs signed rank test.

Figure S4: The Ar inhibitors flutamide and ARN-509 impair cardiac function similarly to PF-998425.

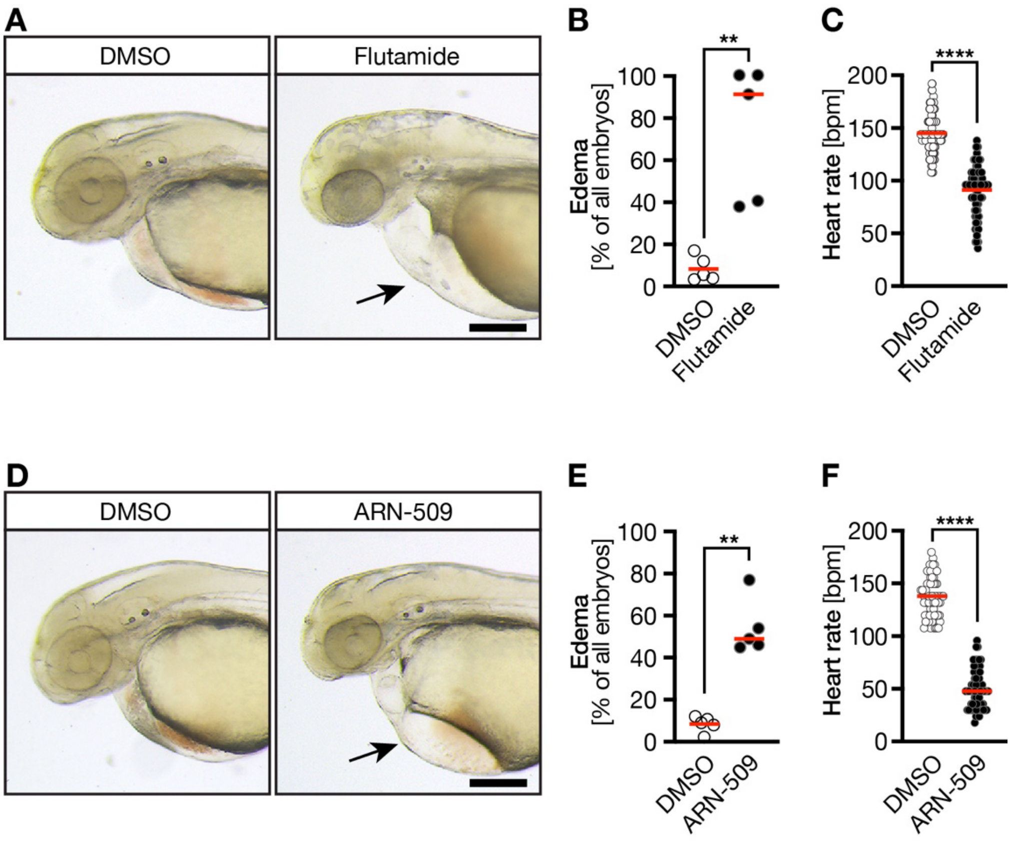

A, Live images of zebrafish treated with vehicle (DMSO) or 25 µM flutamide from tailbud stage on.
B, Edema occurs significantly more often in the flutamide treatment group. n = 5 experiments with 131-137 embryos in total. ** p = 0.0079. Two-tailed Mann-Whitney test.
C, Flutamide-treated embryos display lower heart rates. n = 3 experiments with 138-143 embryos in total. **** p < 0.0001. Two-tailed Mann-Whitney test.
D, Live images of zebrafish treated with vehicle (DMSO) or 50 µM ARN509 from tailbud stage on.
E, Edema formation is increased upon ARN-509 administration. n = 5 experiments with 144-145 embryos in total. ** p = 0.0079. Two-tailed Mann-Whitney test.
F, 50 µM ARN-509 decreases the heart rate. n = 3 experiments with 70-73 embryos in total. **** p < 0.0001. Two-tailed Mann-Whitney test.

Scale bar: 200 µm. Arrows indicate edema.

Figure S5: Ar overexpression produces bradycardia.

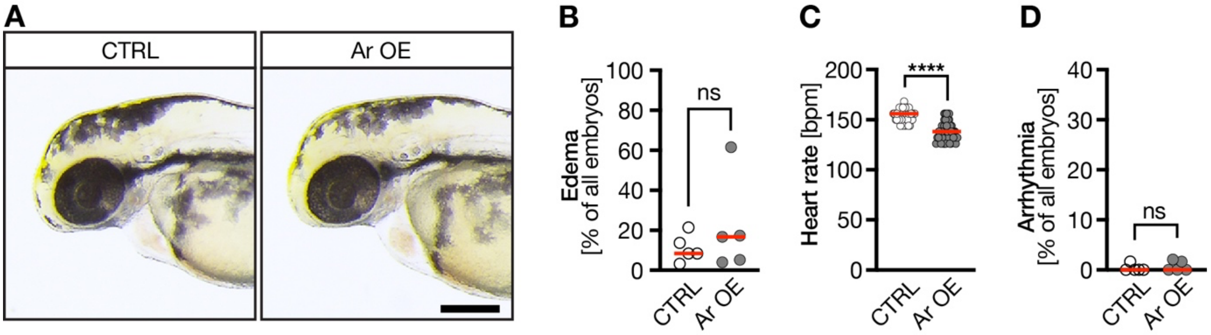

A, Live images of control-injected zebrafish embryos (CTRL) and those injected with capped RNA encoding zebrafish Ar (Ar OE).
B, Graph showing the percentage of embryos displaying edema. n = 5 experiments with 103-104 embryos in total. ns, p = 0.6508. Two-tailed Mann-Whitney test.
C, Ar overexpression decreases the heart rate. n = 3 experiments with 44-51 embryos in total. **** p < 0.0001. Two-tailed Mann-Whitney test.
D, Ar crispants do not display arrhythmia. n = 5 experiments with 103-104 embryos in total. ns, p = 0.7222. Two-tailed Mann-Whitney test.

Scale bar: 200 µm.

Figure S6: Gene editing efficiency of guide RNAs targeting the presumptive promoter of *ar*.

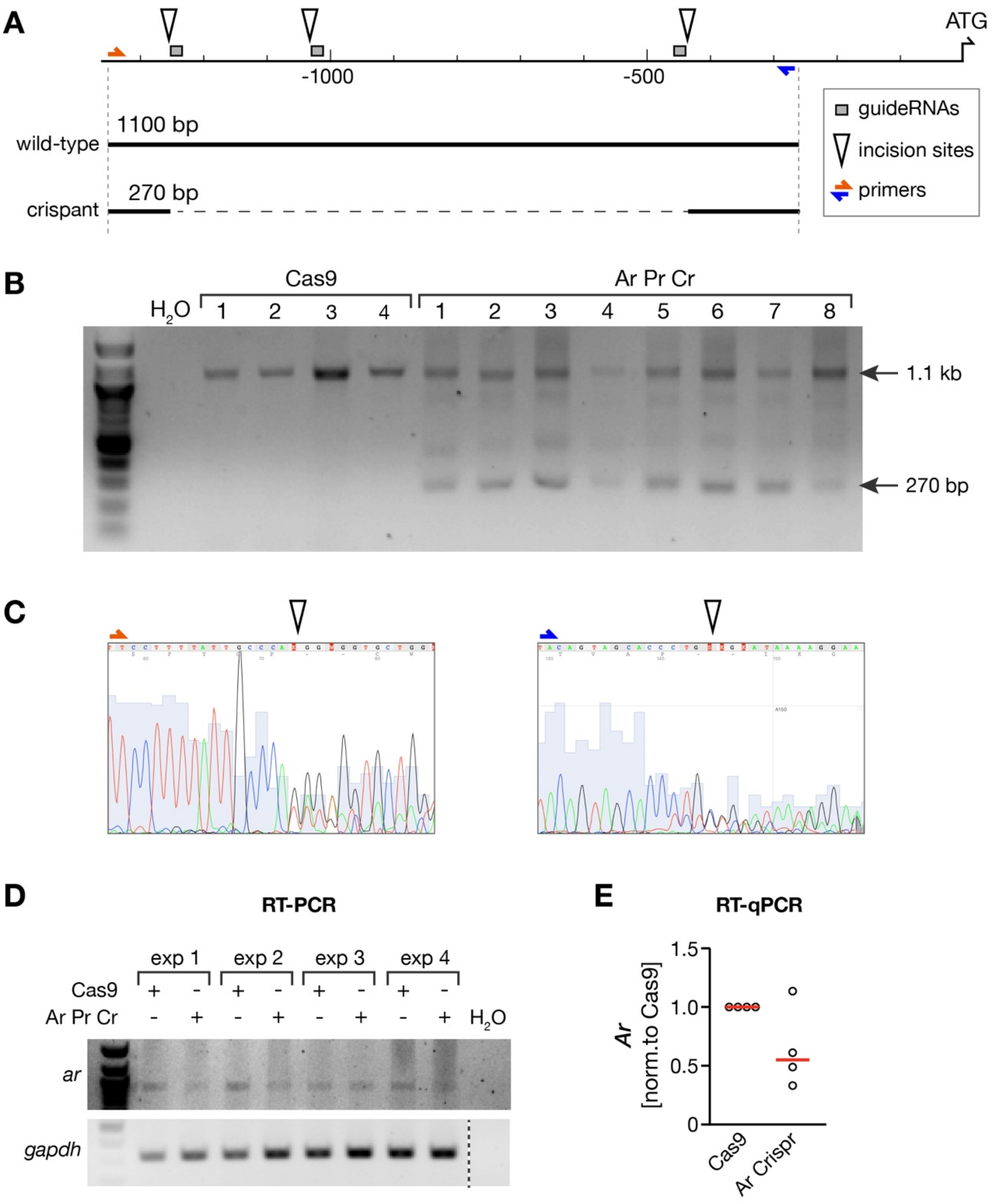

A, Cartoon showing genomic region upstream of start codon of the *ar*, position of the three guideRNAs and of the primers used for PCR.
B, Agarose gel showing PCR to assess gene editing in embryos injected with Cas9 or the ribonucleoprotein complex consisting of all three guideRNAs and Cas9.
C, Example sequencing traces of an Ar Pr Cr PCR using the forward (left panel) and reverse (right panel) primer.
D, RT-PCR shows that Ar promoter crispants express less *ar* RNA. A PCR for *gapdh* was used as loading control.
E, qPCR of the same samples as in **D** showing reduced ar expression in Ar promoter crispants compared to Cas9-injected embryos. Gapdh was used as housekeeping gene

Figure S7: Overall morphology of Ar promoter crispants

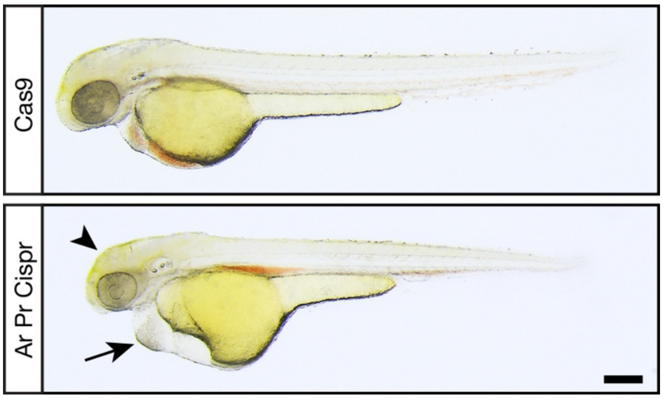

Live images at 48 hpf.
Arrow indicates edema in the pericardiac cavity and the inflow tract. Arrowhead indicates smaller heads.
Scale bar: 200 µm.

Figure S8: Qtc of Ar promoter crispants

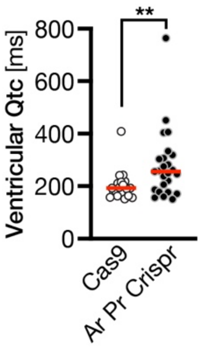

Ar promoter crispants display a longer Qtc than Cas9 embryos at 48 hpf. n = 3 experiments with 19-25 embryos in total. ** p = 0.0082. Two-tailed Mann-Whitney test.

Figure S9: Gene editing efficiency of *ar* guideRNAs targeting exon 1.

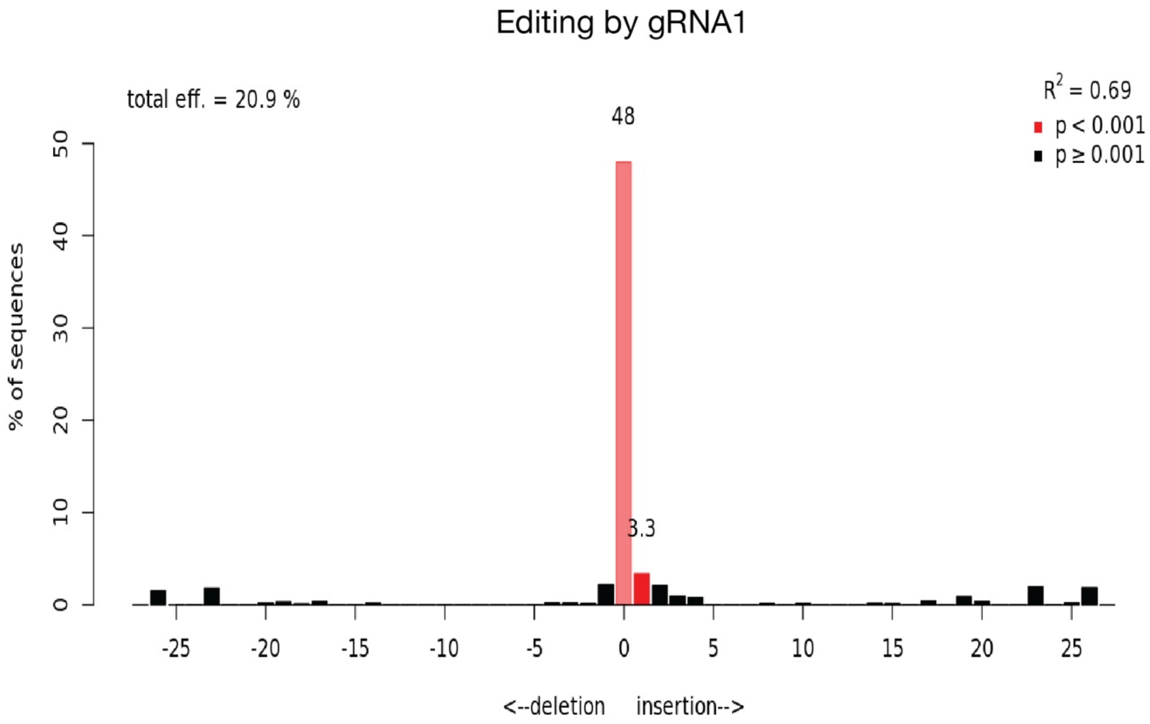

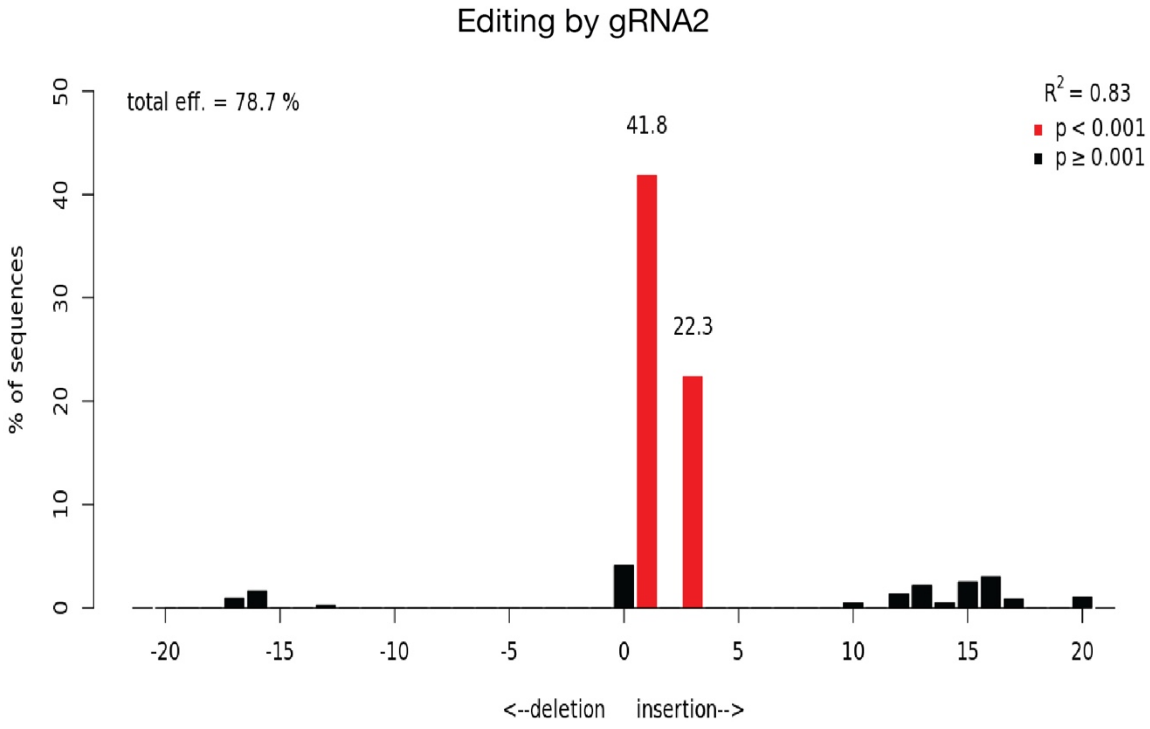

Percentage of gene editing efficiency for the two gRNAs targeted against the *ar*. Results obtained by TIDE (Tracking of Indels by Decomposition) [3].

Figure S10: Ar exon crispants display impaired cardiac function

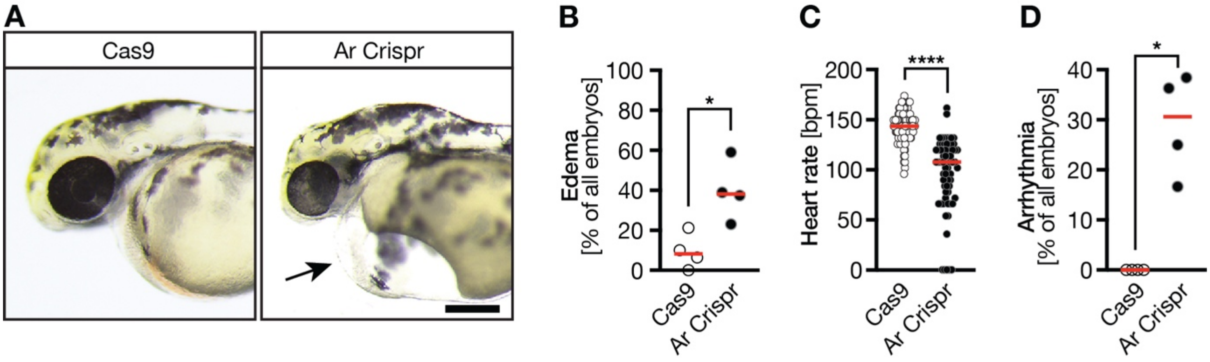

A, Live images of zebrafish embryos injected with Cas9 or Cas9 and three gRNAs against exon 1 of the *ar* (Ar Crispr). Arrow indicates edema.
B, Graph showing the percentage of embryos displaying edema. n = 4 experiments with 69-131 embryos in total. * p = 0.0178. Two-tailed Welch's test.
C, Ar Crispr decreases the heart rate. n = 4 experiments with 69-114 embryos in total. **** p < 0.0001. Two-tailed Mann-Whitney test.
D, Ar crispants display arrhythmia more often than embryos injected with Cas9 only. n = 4 experiments with 69-114 embryos in total. * p = 0.0106. One sample t and Wilcoxon test.

Figure S11: Ar depletion causes a reduction in the size of the cardiac progenitor heart fields.

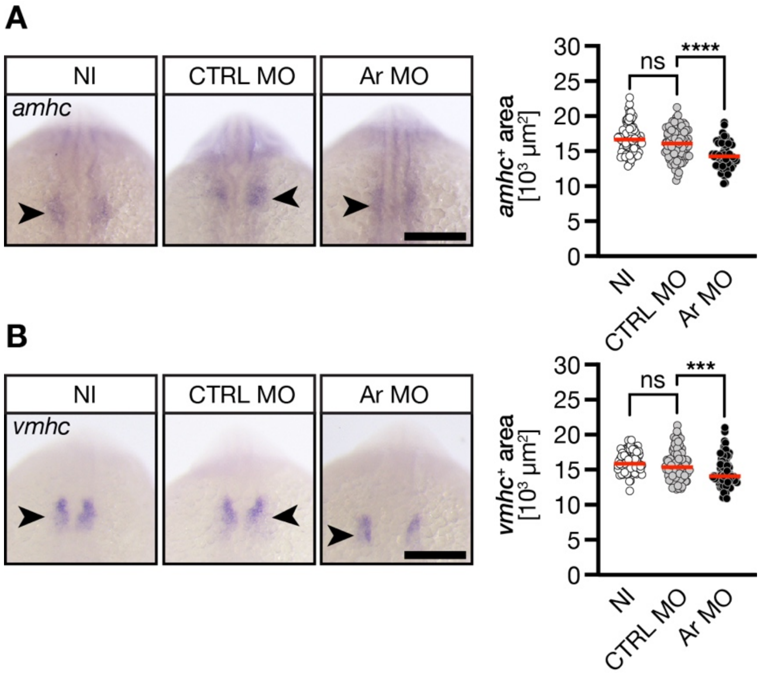

A, WMISH for *atrial myosin heavy chain* (*amhc*) to visualize atrial progenitor cells reveals a small, but significant reduction in atrial heart field size. n = 3 experiments with 51-88 embryos in total. ns, p = 0.0504 (NI vs CTRL MO) and **** p <0.0001 (CTRL MO vs Ar MO). One-way ANOVA with Holm-Sidak's multiple comparisons test.
B, WMISH for *ventricular myosin heavy chain* (*vmhc*) shows that ventricular progenitors are also reduced upon Ar knockdown. n = 3 experiments with 79-99 embryos in total. ns, p = 0.0656, *** p = 0.0009. Kruskal-Wallis test with Dunn's multiple comparison test.

Scale bars: 200 µm. Arrowheads staining in the heart fields. Analyses at 18 somite stage.

Figure S12: Sarcomere structure and contractile gene expression is not altered in Ar LOF embryos.

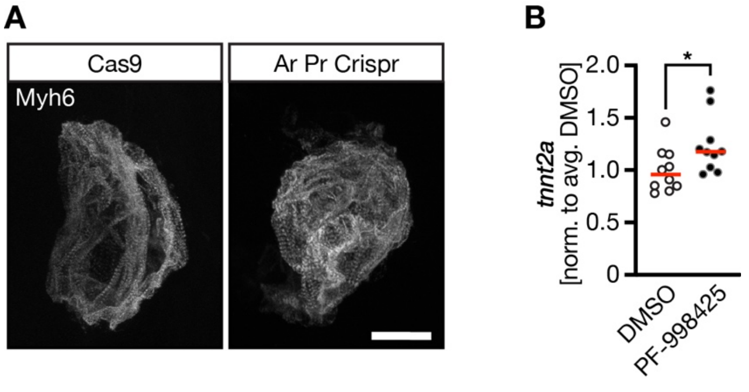

A, Antibody staining of zebrafish hearts. The S46 antibody, which recognizes atrial myosin was used. 5 out 6 Cas9 atria and 4 out of Ar promoter crispants showed the typical sarcomere pattern. Scale bar: 25 µm.
B, qPCR analysis of *tnnt2 (troponin t2a)* expression in DMSO- and PF-998425-treated embryos. n = 10. * p = 0.0288. Two-tailed Mann-Whitney test.

Analyses at 48 hpf stage.

Figure S13: Ejection fraction analysis of embryos upon PF-998425 administration.

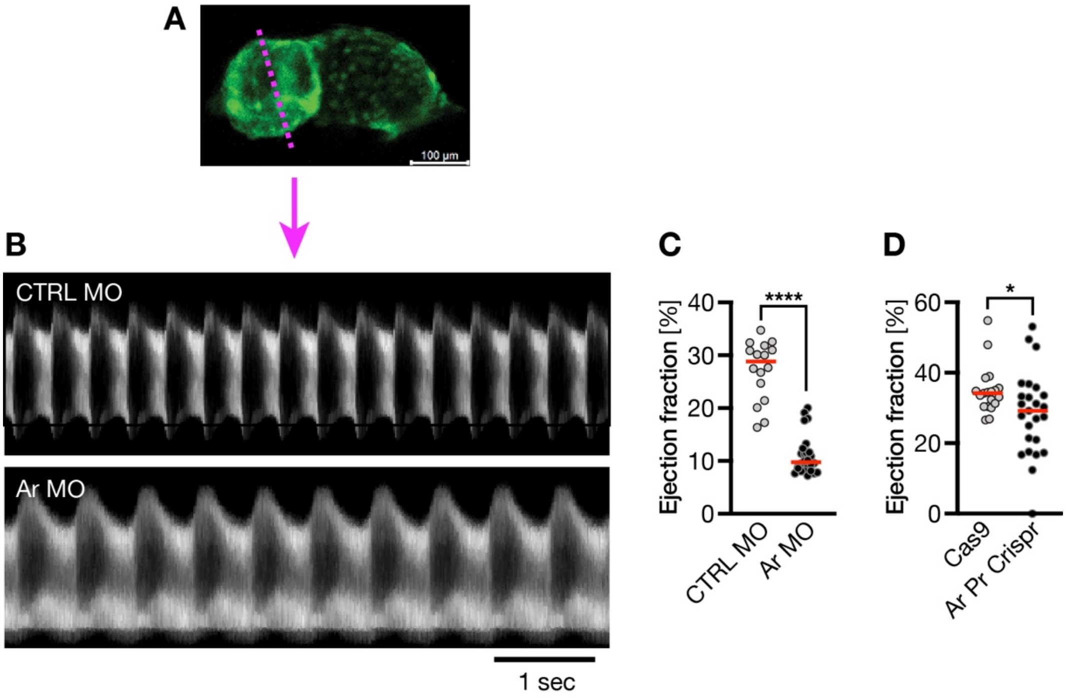

A, Videos of beating hearts expressing GFP in all cardiomyocytes were recorded at 100 frames per second for 10 seconds. Using the Cardiac Performance Software (Viewpoint, France), a line (dashed line in magenta) was drawn through the ventricle, which set the position to generate M-mode-like pictures for measuring the chamber diameter in systole and diastole. Picture shows example heart imaged for ejection fraction analysis.

B, Example M-mode-like pictures (not resembling the whole 10 seconds recording period) generated using the Cardiac performance software. Note, the greatly diminished contractions in the Ar MO as well as the irregularity of the beating.

C, Quantification of ventricular ejection fraction. n = 3 experiments with 17-26 embryos in total. **** $p < 0.0001$. Two-tailed Mann-Whitney test. Outliers were removed using a ROUT test.

D, Ventricular ejection fraction is also reduced in Ar promoter crispants when compared to Cas9 embryos. n = 3 experiments with 19-29 embryos in total. * $p = 0.0248$. Two-tailed Mann-Whitney test.

Figure S14: Representative images of in situ hybridizations for AVC marker genes.

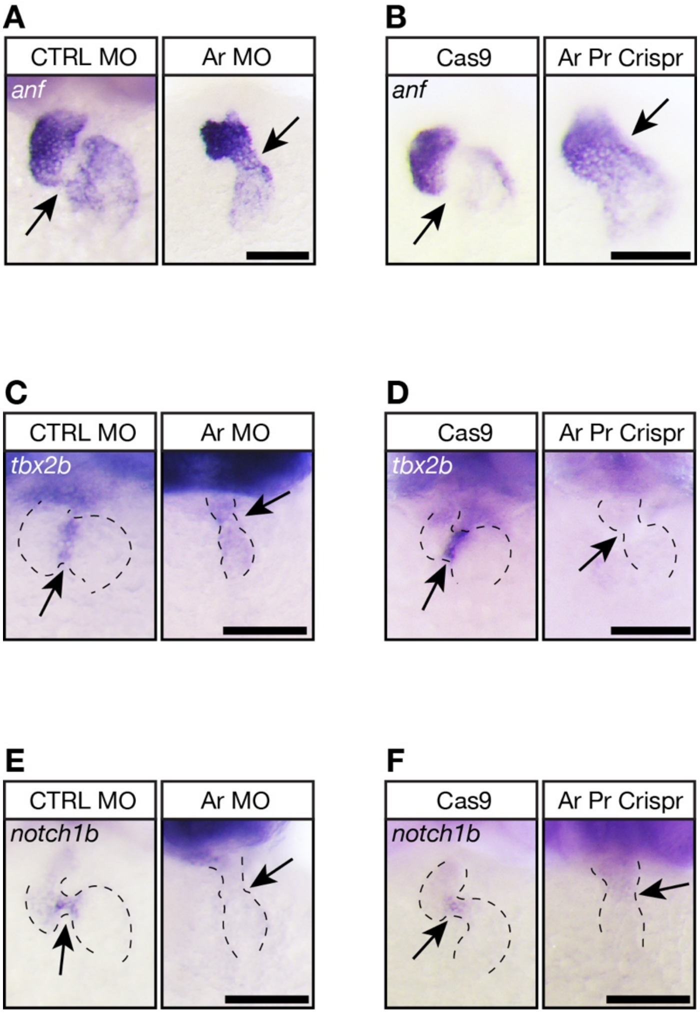

A, *Anf* is excluded from the AVC of CTRL MO-injected embryos, while it is expressed throughout the entire heart including the AVC in Ar morphants.
B, Hearts of Ar promoter crispants express *anf* in the AVC, which cannot be observed in Cas9 control hearts.
C, *Tbx2b* can be detected in the AVC in CTRL MO-injected embryos. Ar morphants display no or only diffuse staining.
D, AVC-restricted *tbx2b* cannot be detected in Ar promoter crispants, but in Cas9-injected embryos.
E, The hearts of CTR MO embryos display a weak *notch1b* signal in the ventricle and clear enrichment in the AVC. Ar morhants fail to accumulate *notch1b* in the AVC.
F, *Notch1b* accumulation can also be observed in hearts of Cas9-injected embryos. In Ar promoter crispants no or only weak ventricular *notch1b* can be detected.

Scale bar: 200 µm. Arrows indicate the AVC. All analyses at 48 hpf stage.

Figure S15: Gene regulation during cardiogenic differentiation of P19 cells.

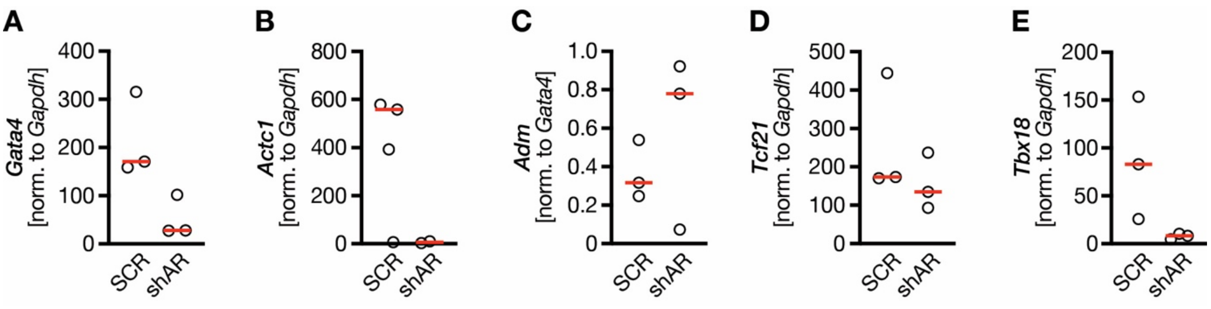

Murine P19 cells were differentiated in the presence of azacytidine towards a cardiogenic fate. At day 10 of differentiation, cells were harvested, RNA isolated and qPCR performed.

SCR, cells stably transfect with a scramble hairpin.
shAR, cells stably transfected with a hairpin against the Ar.

A, As previously reported, cardiac induction as shown by *Gata4* expression is reduced in Ar knockdown cells. n = 3. p = 0.100. Two-tailed Mann-Whitney test.
B, Ar knockdown reduces differentiation into cardiomyocytes as shown by diminished *actin alpha cardiac muscle 1* (*Actc1*) expression. n = 3. p = 0.0134. Two-tailed Welch's test.
C, Compared to *Gata4*, *adrenomedullin* (*Adm*) is upregulated in Ar knockdown cells. n = 3. p = 0.4627. Two-tailed Welch's test
D, There is a tendency towards less expression of the epicardial gene *Tcf21* in Ar knockdown cells. n = 3. p = 0.200. Two-tailed Mann-Whitney test
E, The epicardial marker *Tbx18* is less expressed in cells depleted of the Ar. n = 3. p = 0.2085. Two-tailed Welch's test.

n = 3 experiments. Red line: median. Each circle indicates on differentiation series.

Figure S16: Transient transfection of zebrafish Ar into HEK293T cells.

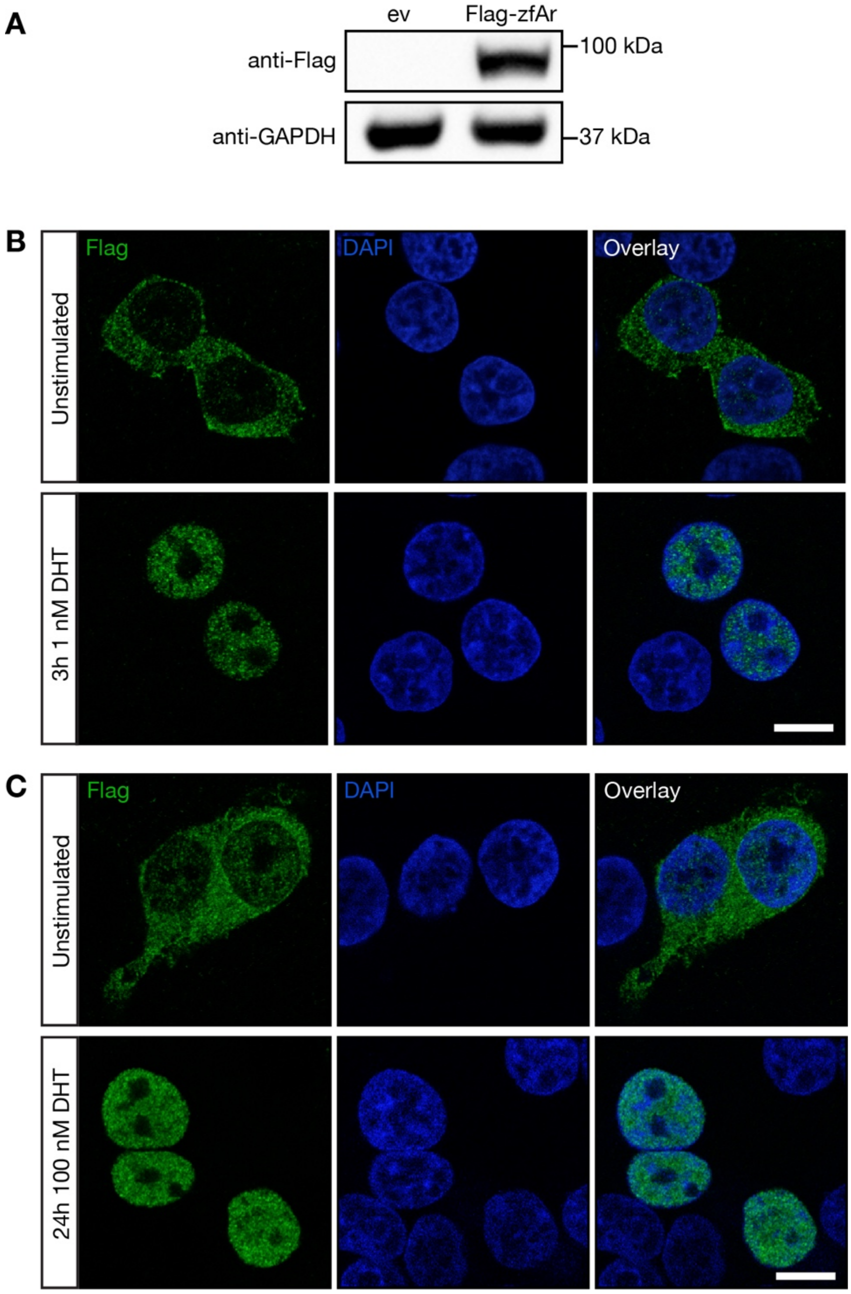

Western blot and immunofluorescence of HEK293T cells transiently transfected with Flag-tagged zebrafish Ar. Antibody staining using anti-Flag (green) antibody.

A, Western blot showing expression of Flag-tagged zebrafish Ar (zfAr) in HEK293T cells. ev, empty vector.
B, Representative images of unstimulated cells and those stimulated for 3 hours with 1 nM Dihydrotestosterone (DHT).
C, Representative images of unstimulated cells and those stimulated for 24 hours with 100 nM Dihydrotestosterone (DHT). Overexpression of zebrafish Ar is sufficient to drive low nuclear localization of the Ar under basal conditions.

Scale bars: 10 µm.

Figure S17: Upregulation of *adm2a* expression.

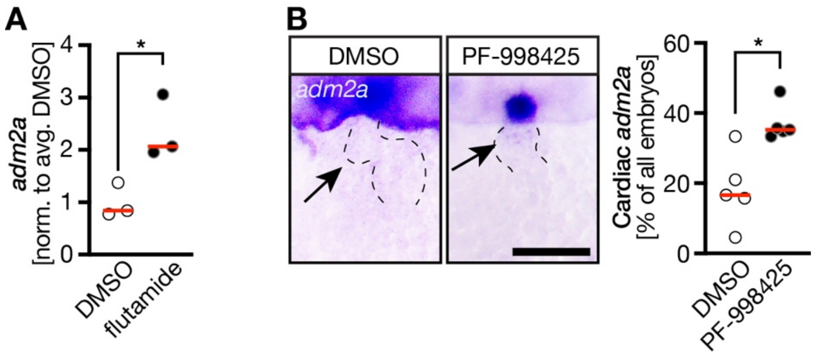

A, Flutamide treatment increases *adm2a* expression as shown by qPCR. n = 3. * p = 0.0400. Two-tailed Welch's test.

B, A*dm2a* expression in the heart (arrow) of Ar LOF embryos is higher than in control embryos. n = 5 experiments with 138-154 embryos. * p = 0.0159. Two-tailed Mann-Whitney test. Scale bar: 200 µm.

Figure S18: The arrhythmogenic compound tolterodine does not induce increased *adm2a* expression.

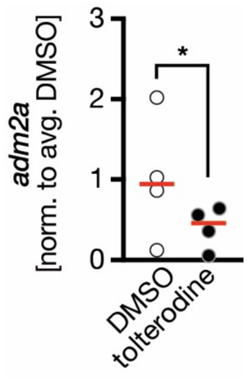

qPCR of 48 hpf zebrafish embryos treated with 10 µM tolterodine. n = 4 experiments. * p = 0.0113, two-tailed ratio paired test. Red line indicates median.

Figure S19: Gene editing efficiency of *adm2a* gRNAs

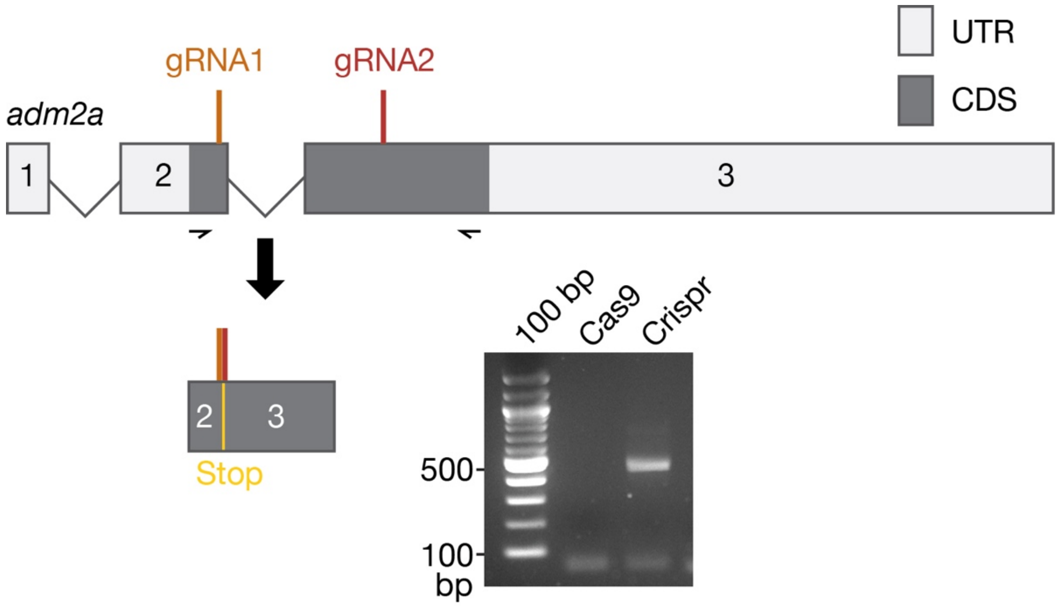

ATG AGA GCG CTT TTC CCG GTT TTT GTG TAT
TGC ATC AGC CTG CTC TCC ACA CAA CTG CTG
GCT CCA CCG TAA CACTCGAGA...

Two gRNAs were selected against sequences in the coding sequence of *adm2a* and injected together in one ribonucleoprotein complex with Cas9. PCR analysis using primers flanking the presumed sites of gene editing revealed deletion of the sequence (including a 9 kb intron) between the two gRNA binding sites resulting in a premature stop codon.

Figure S20: *Receptor (calcitonin) activity modifying protein 2* (*ramp2*) expression at 48 hpf.

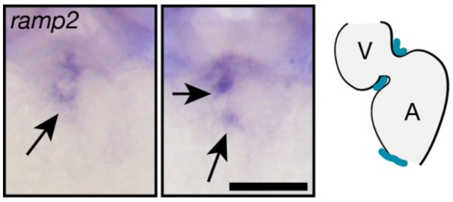

Representative images of WMISH for *ramp2* demonstrating expression in the region of the proepicardium (indicated in blue in the cartoon). Scale bar: 100 μm.

Figure S21: Tcf21 expression at 72 hpf.

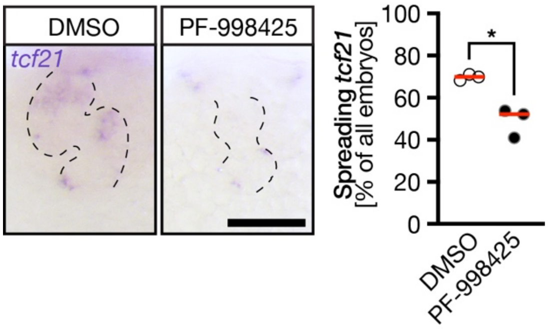

Representative images of WMISH for *tcf21* demonstrating proepicardium gradually spreading over the developing heart.
72 hpf. Scale bar: 100 µm.

n = 3 experiments with 52-86 embryos in total. * $p = 0.0316$. Two-tailed Welch's test.

Figure S22: *Wtip* is reduced upon Ar knockdown.

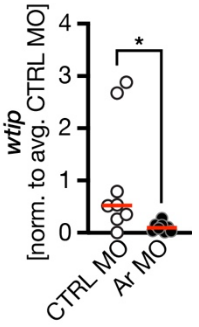

qPCR analysis of *wtip (wilm's tumor interacting protein)* expression in 48 hpf embryos injected with CTRL MO or Ar MO. n = 8. * p = 0.0391. Two-tailed, Wilcoxon matched-pairs signed rank test.

Figure S23: Effects of Bmp2b overexpression.

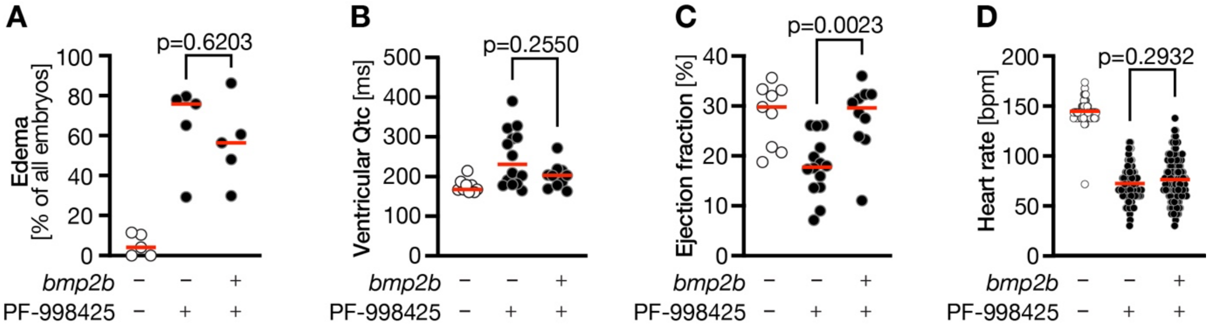

A, Overexpression of Bmp2b possibly improves edema in Ar LOF embryos. n = 5 experiments with 123-142 embryos in total. Kruskal-Wallis test with Dunn's multiple comparisons test.
B, Qtc in PF-998425 embryos and concomitant Bmp2b overexpression. n = 9-14 embryos. Kruskal-Wallis test with Dunn's multiple comparisons test.
C, Improved ejection fraction in PF-998425 embryos in the presence of Bmp2b. n = 9-14 embryos. Brown-Forsythe and Welch ANOVA test with Dunnett's T3 multiple comparisons test.
D, Heart rates upon PF-998425 treatment and Bmp2b overexpression. n = 5 experiments with 123-137 embryos in total. Kruskal-Wallis test with Dunn's multiple comparison test.

All data analyzed at 48 hpf. Red lines: Median. p-values given in the graphs.
Please, note: data in A,B, and D did not follow a normal distribution so that a non-parametric test had to be applied.

SUPPLEMENTARY REFERENCES

1. Pandini G, Mineo R, Frasca F, Roberts CT, Jr., Marcelli M, Vigneri R, et al. Androgens up-regulate the insulin-like growth factor-I receptor in prostate cancer cells. Cancer Res. 2005; 65: 1849-57.

2. Phillippe M, Saunders T, Bangalore S. A mechanism for testosterone modulation of alpha-1 adrenergic receptor expression in the DDT1 MF-2 smooth muscle myocyte. Mol Cell Biochem. 1991; 100: 79-90.

3. Brinkman EK, Chen T, Amendola M, van Steensel B. Easy quantitative assessment of genome editing by sequence trace decomposition. Nucleic Acids Res. 2014; 42: e168.
