# Supplementary material for: Cardiac function in zebrafish embryos is linked to an androgen receptor-adrenomedullin-proepicardium axis
Source: Cell Commun Signal. 2026 Jul 30;24:425. doi: 10.1186/s12964-026-03107-4 (PMC13422332; doi:10.1186/s12964-026-03107-4)
Supplement: Supplementary file 2 — Supplementary Material 2. [file 12964_2026_3107_MOESM2_ESM.pdf]

Duong Phu et al, Unedited blot and gel images

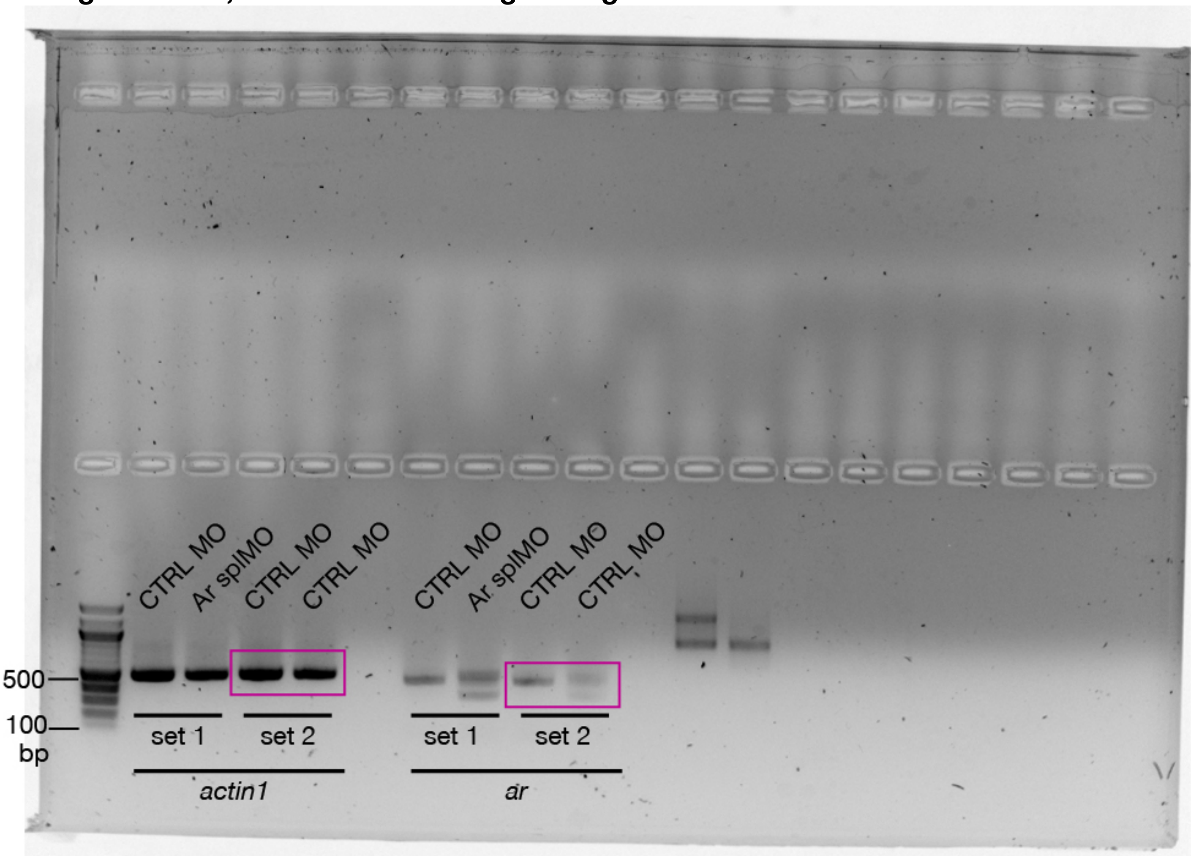

Unedited gel image related to Figure 1E.
Bp, basepairs.

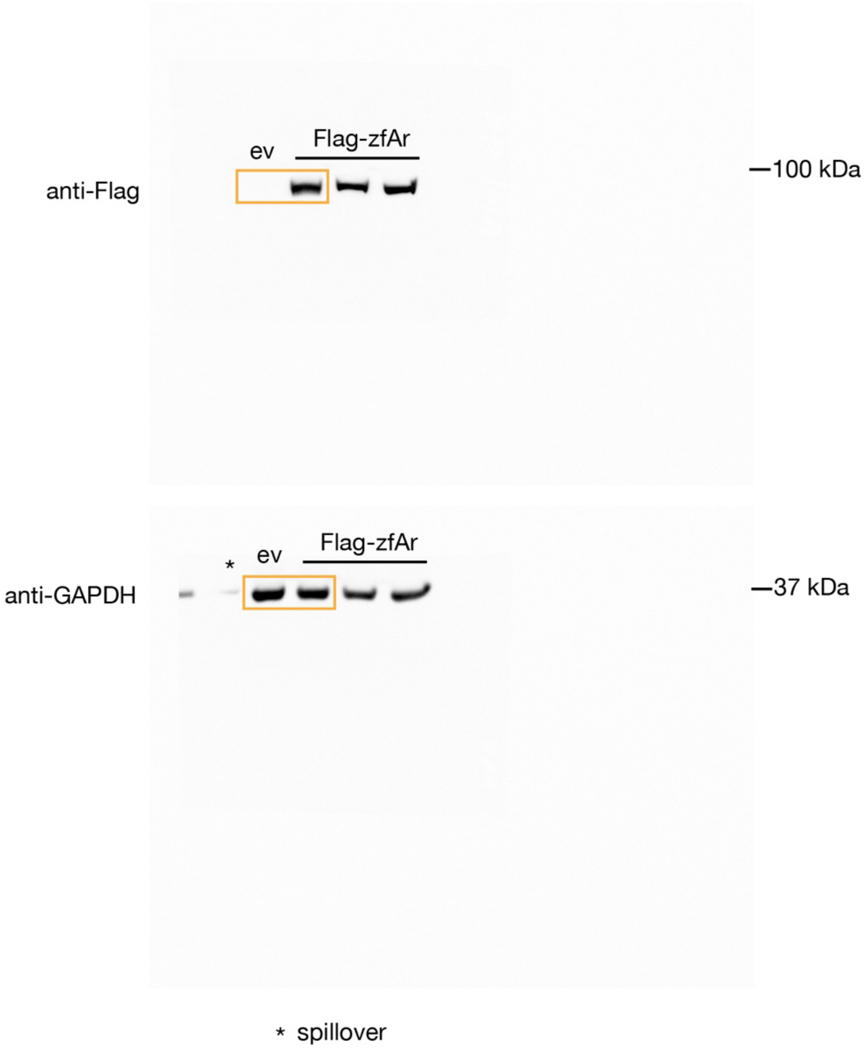

Unedited Western blots related to Figure S16.
ev, empty vector
