# Supplementary figures and images for: Cardiac function in zebrafish embryos is linked to an androgen receptor-adrenomedullin-proepicardium axis
Source: Cell Commun Signal. 2026 Jul 30;24:425. doi: 10.1186/s12964-026-03107-4 (PMC13422332; doi:10.1186/s12964-026-03107-4)

## Slide 1
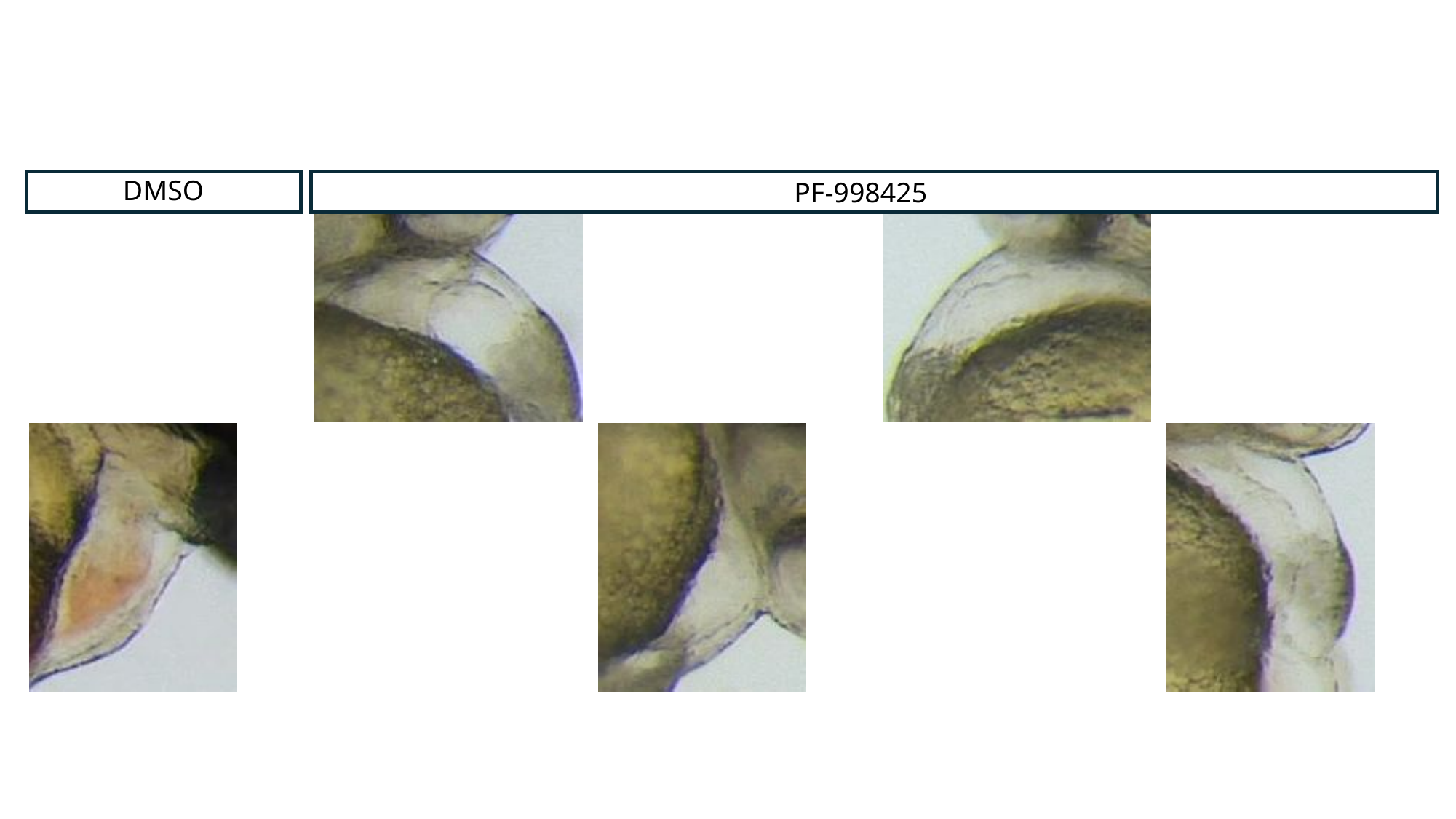

DMSO
PF-998425

Supplement: Supplementary file 3 — Supplementary Material 3. [file 12964_2026_3107_MOESM3_ESM.pptx]

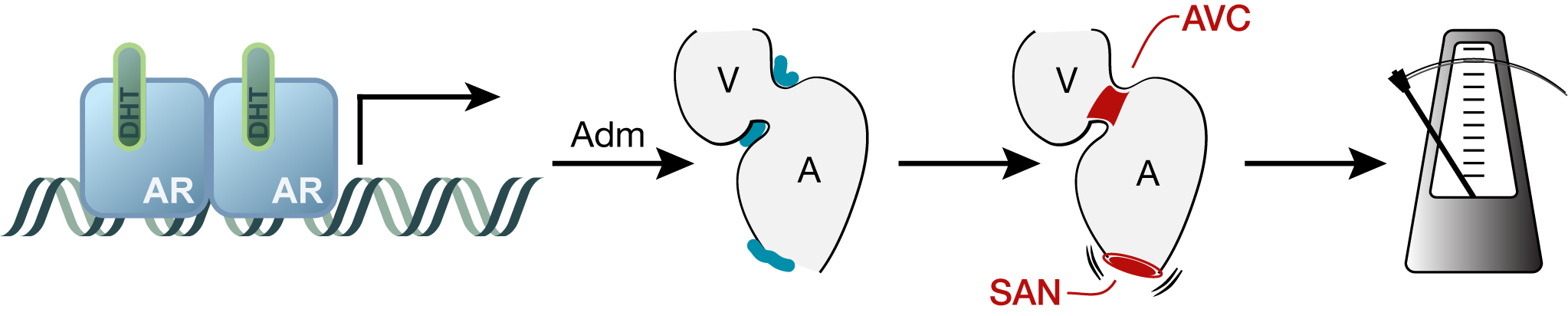

Supplement: Supplementary file 4 — Supplementary Material 4. [file 12964_2026_3107_MOESM4_ESM.tif]
